# A Multidimensional Investigation of Sensory Processing in Autism: Parent- and Self-Report Questionnaires, Psychophysical Thresholds, and Event-Related Potentials in the Auditory and Somatosensory Modalities

**DOI:** 10.3389/fnhum.2022.811547

**Published:** 2022-05-10

**Authors:** Patrick Dwyer, Yukari Takarae, Iman Zadeh, Susan M. Rivera, Clifford D. Saron

**Affiliations:** ^1^Neurocognitive Development Lab, Center for Mind and Brain, University of California, Davis, Davis, CA, United States; ^2^Department of Psychology, University of California, Davis, Davis, CA, United States; ^3^MIND Institute, University of California, Davis, Davis, CA, United States; ^4^Department of Neurosciences, University of California, San Diego, San Diego, CA, United States; ^5^Department of Psychology, San Diego State University, San Diego, CA, United States; ^6^Oracle Cloud Infrastructure, Oracle Corporation, Los Angeles, CA, United States; ^7^Saron Lab, Center for Mind and Brain, University of California, Davis, Davis, CA, United States

**Keywords:** autism, sensory, auditory, tactile, event-related potentials, psychophysics

## Abstract

**Background:**

Reconciling results obtained using different types of sensory measures is a challenge for autism sensory research. The present study used questionnaire, psychophysical, and neurophysiological measures to characterize autistic sensory processing in different measurement modalities.

**Methods:**

Participants were 46 autistic and 21 typically developing 11- to 14-year-olds. Participants and their caregivers completed questionnaires regarding sensory experiences and behaviors. Auditory and somatosensory event-related potentials (ERPs) were recorded as part of a multisensory ERP task. Auditory detection, tactile static detection, and tactile spatial resolution psychophysical thresholds were measured.

**Results:**

Sensory questionnaires strongly differentiated between autistic and typically developing individuals, while little evidence of group differences was observed in psychophysical thresholds. Crucially, the different types of measures (neurophysiological, psychophysical, questionnaire) appeared to be largely independent of one another. However, we unexpectedly found autistic participants with *larger* auditory Tb ERP amplitudes had *reduced* hearing acuity, even though all participants had hearing acuity in the non-clinical range.

**Limitations:**

The autistic and typically developing groups were not matched on cognitive ability, although this limitation does not affect our main analyses regarding convergence of measures within autism.

**Conclusion:**

Overall, based on these results, measures in different sensory modalities appear to capture distinct aspects of sensory processing in autism, with relatively limited convergence between questionnaires and laboratory-based tasks. Generally, this might reflect the reality that laboratory tasks are often carried out in controlled environments without background stimuli to compete for attention, a context which may not closely resemble the busier and more complex environments in which autistic people’s atypical sensory experiences commonly occur. Sensory questionnaires and more naturalistic laboratory tasks may be better suited to explore autistic people’s real-world sensory challenges. Further research is needed to replicate and investigate the drivers of the unexpected association we observed between auditory Tb ERP amplitudes and hearing acuity, which could represent an important confound for ERP researchers to consider in their studies.

## Introduction

Increasing evidence highlights the importance of atypical sensory processing within the autistic phenotype. Not only can atypical sensory processing emerge early in development and predict later social features of Autism Spectrum Development (ASD; Baranek, 1999; Baranek et al., 2018; Damiano-Goodwin et al., 2018; Kolesnik et al., 2019), but sensory processing is a correlate (Lin and Huang, 2019) or aspect (McConachie et al., 2020) of autistic people’s quality of life.^1^ However, different sensory measures such as questionnaires, psychophysical thresholds, and neurophysiological responses frequently appear to yield radically different patterns of ASD-Typical Development (TD) group differences.

Prior studies using both parent- and self-report questionnaires to examine different patterns of atypical sensory processing, such as hyposensitivity, hypersensitivity, and sensory interests, generally report robust differences between autistic and typically developing individuals (Kientz and Dunn, 1997; Baranek et al., 2006; Tomchek and Dunn, 2007; Crane et al., 2009; Takayama et al., 2014; Tavassoli et al., 2014).

Psychophysical studies appear markedly less likely than questionnaire studies to find ASD-TD differences, and what differences are observed may be specific to particular modalities, tasks, or subgroups. In the tactile domain, some prior research using static stimuli (such as Von Frey monofilaments) suggests touch detection thresholds do not differ between autistic and typically developing individuals (Cascio et al., 2008; Fukuyama et al., 2017), and while other findings suggest poorer tactile static detection thresholds in ASD (Puts et al., 2014), this might reflect conservative perceptual decision-making and reporting (Quinde-Zlibut et al., 2020). Some studies suggest detection thresholds for vibrotactile stimuli could be enhanced in ASD (Blakemore et al., 2006; Cascio et al., 2008; Sapey-Triomphe et al., 2019), but this effect may depend on frequency and body site (Blakemore et al., 2006; Cascio et al., 2008) and is not universally reported (Puts et al., 2014; Ide et al., 2019). Autistic and typically developing groups might not differ in hearing acuity (Khalfa et al., 2004; Demopoulos and Lewine, 2016; Kuiper et al., 2019) or might exhibit only modest differences of variable directionality across frequencies (Gravel et al., 2006). However, enhanced pitch discrimination has been reported in subgroups within ASD (Jones et al., 2009; Bonnel et al., 2010; see also Mayer et al., 2016).

Event-related potentials (ERPs) and event-related fields (ERFs) have been widely used to investigate auditory processing in ASD (Haesen et al., 2011; O’Connor, 2012; Williams et al., 2021a). However, such studies can be complicated by developmental change: in TD, at 9–14 years of age, overlapping with the age range of the present study, the child frontocentral P1-N2 complex evolves into the adult central P1-N1-P2-N2 (see Sharma et al., 1997; Albrecht et al., 2000; Ponton et al., 2002; Gilley et al., 2005). Auditory temporal Tb (also called N1c; in contrast to the aforementioned central N1 or N1b) responses appear more stable over this age range (Albrecht et al., 2000; Ponton et al., 2002); the Tb is a negative-going response over temporal sites around ∼150 ms after auditory stimulus onset. Another response that appears to be stable over these years is an early somatosensory ERP, a contralateral centro-parietal positivity occurring around ∼55 ms (Uppal et al., 2016) which might correspond to the adult P60 (see, e.g., Eimer et al., 2002; Schubert et al., 2006; Pratt, 2011). Prior research reports that Tb amplitudes are attenuated in ASD (Williams et al., 2021a); in addition, the small number of prior studies examining Tb latencies have reported delays (Williams et al., 2021a). In contrast, ASD-TD group differences in frontal N1 amplitudes and latencies appear relatively modest or non-significant (Williams et al., 2021a). Furthermore, somatosensory responses in the P60 time window may be reduced in amplitude in ASD (Russo et al., 2010; Marco et al., 2012).

In a recent review of the field, Uljarević et al. (2017) identify discrepancies between different types of sensory measures as a major challenge for research. These authors suggest that sensory features should be assessed using multiple approaches.

Theories informing the design of widely-used sensory questionnaires, such as the Sensory Profile (SP) and Adolescent/Adult Sensory Profile (AASP), suggest that some form of stimulus detection or behavioral reaction threshold is captured by these measures (Dunn, 1997). However, prior autism studies suggest a lack of robust, consistent associations between tactile thresholds and sensory questionnaire scores across multiple modalities (Ide et al., 2019; Quinde-Zlibut et al., 2020). Research in the general population also fails to find associations between hearing thresholds and sensory questionnaire scores (Schulz and Stevenson, 2021). That being said, elevated hearing thresholds and poorer intensity discrimination in ASD might be related to questionnaire reports of greater sensitivity and avoidance (Jones et al., 2009; Kuiper et al., 2019). Tactile temporal resolution might show similar patterns (Ide et al., 2019).

There are considerable differences between the sensory processing in an individual’s natural environment and in psychophysical laboratory tasks. In natural environments, individuals are often exposed to many stimuli simultaneously. Attention capture (to which autistic people may be suspectible; see, e.g., Keehn et al., 2016; Remington and Fairnie, 2017; Venker et al., 2021) by aversive stimuli in these busy environments might lead to experiences of sensory distress and overload. Participants completing sensory questionnaires are presumably reporting these real-world experiences. In contrast, psychophysical tasks provide participants with clear attention instructions in controlled, low-stimulation environments. In the absence of distractors capable of capturing or overwhelming attention, autistic and non-autistic individuals might process stimuli relatively similarly.

Some prior studies have also examined, in ASD, convergence between questionnaire reports of sensory processing and ERP/ERF responses to auditory stimuli. For example, larger early neural responses to sounds could be related to reports of auditory distractibility or sensory sensitivity (Karhson and Golob, 2016; Dwyer et al., 2020). Prior research has also explored associations between questionnaire-reported sensory behaviors and auditory mismatch/P3 responses (Ludlow et al., 2014; Chien et al., 2017) as well as ERP indices of auditory habituation (Hudac et al., 2018). Weaker neural responses to air puffs, relative to sham, appear to correlate with reports of tactile hypo-responsiveness, and at a trend level, larger neural responses may be related to reported tactile hyper-responsiveness (Cascio et al., 2015). Studies have also reported associations between ERPs and observational measures of sensory processing (Donkers et al., 2020; Schwartz et al., 2020a).

It appears intuitively reasonable to imagine that questionnaire reports of hyper- or hypo-responsivity to stimuli should be related to neural hyper- and hypo-responsiveness, especially when brain responses are recorded in a context comparable to the busy real world. Indeed, there may be associations between ERPs and sensory questionnaire scores when ERPs are recorded in passive paradigms while background stimuli compete for attention (as in Dwyer et al., 2020; Schwartz et al., 2020b). However, convergence between sensory questionnaire scores and ERP amplitudes has also been observed while participants complete active tasks, requiring maintenance of particular attentional sets, in low-stimulation environments (e.g., Karhson and Golob, 2016), and conversely, studies using passive paradigms do not always report clear associations between ERPs and sensory questionnaire scores (e.g., Donkers et al., 2015).

There is currently minimal research regarding associations between neurophysiological responses and psychophysical thresholds in ASD. One study reports no associations between latency of somatosensory ERFs and tactile spatial resolution or tactile proprioception (Demopoulos et al., 2017). It is therefore tempting to speculate that there would be little direct relationship between neurophysiological responses to a clear, suprathreshold stimulus and the ability to detect a much subtler, near-threshold stimulus, but further research is needed.

The present study examines sensory processing in ASD using a mixture of physiological, behavioral/psychophysical, and questionnaire measures in well-characterized groups of autistic and typically developing children. This rich, multimethod dataset allows the present study to compare the results obtained with different methods and to determine whether they converge or appear to reflect distinct aspects of sensory processing.

Hypotheses:

(1) Autistic individuals will, in comparison to typically developing participants, be reported on questionnaires to exhibit more atypical sensory processing in all domains (e.g., more hyper- and hypo-sensitivity, more sensory interests, more enhanced perception), in keeping with prior research;
(2) Autistic and typically developing individuals will not differ from one another in hearing acuity, tactile detection thresholds, or tactile spatial resolution thresholds;
(3) Amplitudes of the auditory Tb and somatosensory P60 ERPs will be attenuated in the ASD group, and their latencies will be delayed; and
(4) Auditory Tb and somatosensory P60 amplitudes in the ASD group will not be associated with hearing and tactile psychophysical thresholds.

## Materials and Methods

### Participants

Participants were recruited through a mixture of community advertising and extant research contact databases, including the UC Davis Health MIND Institute Research Volunteer Registry. 46 autistic (sex: 41 male, 5 female) and 21 typically-developing participants (sex: 14 male, 7 female) provided usable data on at least one questionnaire, psychophysical, or ERP measure and were included in the present study (Table 1). Twenty-six participants (13 autistic, 13 typically developing) provided usable data on all measures (including all questionnaire subscales).

**TABLE 1:** Characteristics of autistic and typically-developing participants.

	ASD			TD			*p*	δ
	Mean (*SD*)	Range	*n*	Mean (*SD*)	Range	*n*		
Chronological Age (years)	12.73 (1.17)	11.05–14.97	46	13.04 (0.93)	11.57–14.72	21	0.24	–0.18
WISC Full-Scale IQ (FSIQ)	97.37 (18.26)	60–125	46	122.14 (11.09)	91–139	21	<0.0001	–0.76
WISC Verbal Comprehension Index (VCI)	100.89 (19.71)	59–134	46	126.24 (11.62)	99–152	21	<0.0001	–0.74
WISC Perceptual Reasoning Index (PRI)	106.33 (16.33)	67–143	46	120.52 (15.21)	84–141	21	0.001	–0.50
WISC Working Memory Index (WMI)	92.70 (16.72)	52–126	46	108.33 (10.19)	91–126	21	<0.0001	–0.61
WISC Processing Speed Index (PSI)	86.46 (16.40)	53–126	46	107.62 (11.87)	91–131	21	<0.0001	–0.70
SCQ Total	22.51 (5.29)	11–35	43	1.10 (1.55)	0–5	21	<0.0001	1.00
ASSQ Total	29.48 (8.43)	12–45	42	1.00 (1.73)	0–7	21	<0.0001	1.00
ADOS Total CSS	6.66 (1.72)	3–10	44	–	–	0	–	–
ADOS Social Affect CSS	6.25 (1.99)	3–10	44	–	–	0	–	–
ADOS “Restricted and Repetitive Behaviors” CSS	7.41 (2.33)	1–10	44	–	–	0	–	–
ADI-R Social Interaction	20.82 (4.19)	13–30	38	–	–	0	–	–
ADI-R Communication	17.61 (3.82)	7–25	38	–	–	0	–	–
ADI-R “Restricted and Repetitive Behaviors”	7.95 (2.23)	4–12	38	–	–	0	–	–

*Mean and standard deviation (SD) are given on each metric, along with minimum and maximum scores. The numbers of participants in each group with available data on each metric are also reported. Where measures were collected from participants in both the ASD and TD groups, Wilcoxon–Mann–Whitney rank-sum tests are used to compare scores across groups; δ (Cliff, 1993) is reported as an effect size. ADI-R scores are based on the diagnostic algorithm, not the current behavior algorithm. For reference, ASD cut-offs are 15 on the SCQ total score, 4 on the ADOS Calibrated Severity Scores (CSS), 10 on the ADI-R Social Interaction score, 8 on the ADI-R Communication score (for verbal participants like those in the present study), and 3 on the ADI-R “Restricted and Repetitive Behaviors” score (Lord et al., 1994; Berument et al., 1999; Gotham et al., 2009). The developers of the ASSQ parent-report form recommend 13 as a sensitive cut-off score and 19 as a specific cut-off score (Ehlers et al., 1999).*

All included participants were required to have Wechsler Intelligence Scale for Children-IV (WISC-IV; Wechsler, 2003) Perceptual Reasoning Index (PRI) scores of at least 65. The PRI index was chosen as a basis for this inclusion criterion as its subtests impose few percepto-motor demands, and this could make the PRI index a more suitable measure of fluid cognitive ability in ASD than other WISC-IV indices (Nader et al., 2015, 2016). Exclusionary criteria for both the ASD and TD groups included a history of non-febrile seizures, a history of serious head trauma, use of antipsychotic or barbiturate medications, known hearing loss, and known visual impairment.

The autism spectrum diagnoses of 43 autistic participants were verified by clinical judgment and using the Autism Diagnostic Observation Schedule (ADOS; Lord et al., 2000); all of these participants met “autism” or “autism spectrum” criteria per the revised algorithms published by Gotham et al. (2007) and Hus and Lord (2014). One further autistic participant fell a point short of ADOS criteria, but this participant did meet autism criteria per the Autism Diagnostic Interview-Revised (ADI-R; Lord et al., 1994) diagnostic algorithm and clinical judgment suggested that they met DSM-IV diagnostic criteria for a pervasive developmental disorder. The remaining two autistic participants’ diagnoses were supported by a recent (<1.25 years) external diagnostic evaluation that included administration of the ADOS.

The parent-report Social Communication Questionnaire (SCQ; Berument et al., 1999) and Autism Spectrum Screening Questionnaire (ASSQ; Ehlers et al., 1999) were used to screen typically developing participants for autism. Exclusion criteria for the TD group included parent reports of a history of developmental, learning, or genetic conditions or neurodivergence; as well as first-degree genetic relatives with known autism spectrum diagnoses.

### Sensory Questionnaire Measures

#### Adolescent/Adult Sensory Profile

The AASP is a self-report questionnaire with 60 items measuring the frequency of sensory behaviors and experiences using a 5-point Likert scale (Brown and Dunn, 2002). It is based on the model of sensory processing proposed by Dunn (1997), which describes sensory processing in terms of (1) neurological thresholds, understood in terms of variation in the amount/intensity of a stimulus needed for detection and registration to occur, and (2) either behaving in accordance with a threshold or “counteracting” thresholds by seeking or avoiding stimuli. As a result, the AASP provides four “quadrant” scores reflecting the interaction of thresholds and behavioral responding:

• *Low registration*: High thresholds (i.e., poor detection of stimuli) and passive responding (i.e., despite low stimulation, not attempting to seek stimuli).
• *Sensory seeking*: High thresholds and counteracting of high thresholds through stimulation-seeking.
• *Sensory sensitivity*: Low thresholds (i.e., enhanced detection of stimuli) and behavioral responding in accordance thresholds (e.g., becoming distracted by incoming stimuli).
• *Sensory avoiding*: Low thresholds and counteracting of these thresholds through avoiding stimulation.

The AASP, which was developed for use with individuals aged 11 years or older, examines these quadrants through items relating to the taste/smell, movement, visual, touch, activity, and auditory modalities.

Given the present study’s focus on comparing different types of measure, we focused some analyses on questionnaire subscales and items that one might expect to be particularly relevant to auditory and tactile psychophysical thresholds and ERPs.

For the purpose of examining correlations between AASP scores and psychophysical thresholds, because Dunn (1997)’s quadrant model suggests that both sensory sensitivity and sensory avoiding reflect lower thresholds, we summed these items from the auditory (six items) and tactile (seven items) modalities to produce AASP “Low Threshold” scores for each modality.

For analyses of ASD-TD group differences, and of correlations between AASP scores and ERPs, we summed scores in all relevant modality-quadrant combinations separately: i.e., auditory low registration (three items), auditory sensation seeking (two items), auditory sensory sensitivity (three items), auditory sensation avoiding (three items), tactile low registration (three items), tactile sensation seeking (three items), tactile sensory sensitivity (four items), and tactile sensation avoiding (three items). This structure is most similar to that used in our Sensory Experiences Questionnaire (SEQ) analysis, described below.

In the present study, usable AASP data were obtained from 34 autistic and 18 typically developing participants. Twelve missing item responses (0.38%) were imputed using DataWig, a machine learning Python package (Bießmann et al., 2019).

#### Sensory Experiences Questionnaire-3.0

The Sensory Experiences Questionnaire Version 3.0 (SEQ-3.0) is a caregiver-report questionnaire with 97 items measuring the frequency of sensory behaviors using a 5-point Likert scale (Ausderau et al., 2014). SEQ-3.0 items examine the auditory, visual, tactile, gustatory/olfactory, and vestibular/proprioceptive modalities as well as social and non-social contexts. As reported by Ausderau et al. (2014) the SEQ’s items also canonically load onto the four sensory response pattern factors of hypo-responsiveness (HYPO), hyper-responsiveness (HYPER), sensory interests, repetitions, and seeking (SIRS), and enhanced perception (EP). However, Williams et al. (2021b) report that these four canonical sensory response patterns explain relatively limited variance; these authors instead recommend that single-modality response patterns (e.g., tactile HYPER, visual SIRS) should be used to report and analyze findings.

Given the present study’s focus on auditory and tactile modalities, we calculated auditory HYPO (3 items), auditory HYPER (four items), auditory EP (four items), auditory SIRS (two items), tactile HYPO (four items), tactile HYPER (eleven items), and tactile SIRS (seven items) scores. As the SEQ has only a single EP item from the tactile modality (regarding skill identifying unseen objects in bags), we omitted that modality-pattern combination.

Although the SEQ-3.0 was developed for children in the age range of 2–12 years, it has the advantage of having been specifically designed with a focus on autism (Baranek et al., 2006; Ausderau et al., 2014). In the present study, usable SEQ data were obtained regarding 44 autistic and 21 typically developing participants. Twenty-seven missing item responses (0.43%) were imputed using DataWig (Bießmann et al., 2019).

#### Sensory Profile

The Sensory Profile is a caregiver-report questionnaire with 125 items measuring the frequency of sensory behaviors (Dunn, 1999), originally developed for use with children aged 3–10. Like the AASP, it is based on the quadrant model developed by Dunn (1997); however, the SP has a total of nine factors rather than the four quadrants in the AASP. Unfortunately, per Dunn (1997), relatively few auditory and tactile items are included in these nine canonical factors.

Fortunately, the SP distinguishes between “high” and “low” threshold items. To restrict our analyses to the auditory and tactile modalities, we summed low threshold auditory (items 1–5) and tactile items (items 29–39) to produce SP “Low Threshold” scores for each modality. We also calculated “High Threshold” scores for auditory (items 6–8) and tactile (items 40–46) items.

In the present study, SP data from all modalities were obtained regarding 34 autistic and 19 typically-developing participants; another 4 autistic and 1 typically-developing participants had responses from some modalities but not others. Twelve missing item responses (0.18%) were imputed using DataWig (Bießmann et al., 2019).

Data from all modalities on all three questionnaires (AASP, SP, and SEQ) were obtained regarding 30 autistic and 17 typically developing participants.

### Sensory Behavioral/Psychophysical Measures

#### Audiometry

An Otovation Amplitude T4 clinical audiometer and headphone system was used to measure monaural pure tone auditory detection thresholds while participants were seated in a dimly-lit, shielded, audiometrically quiet chamber. Participants were instructed to press a button whenever they heard a tone, even if it was really soft or if they just thought they might have heard a tone. Stimuli were 1000 ms pure tones of 125, 250, 500, 1000, 2000, 4000, and 8000 Hz, presented at a random interstimulus interval of 1200–2500 ms. Starting intensity level was set at 40 dB HL and levels were either increased in steps of 5 dB or decreased in steps of 10 dB using an automated implementation of the Hughson-Westlake method. The minimum possible intensity was -10 dB HL. One participant with monaural clinical hearing loss (pure tone average (PTA) ≥ 20 dB) was excluded, after which usable audiometric data remained from a total of 40 autistic and 19 typically-developing participants.

#### Tactile Static Detection

Static Von Frey monofilaments (Stoelting Co, 2001), also referred to as Semmes-Weinstein monofilaments, were used to estimate tactile static detection thresholds. Participants were seated at a table in front of a large, opaque cardboard folding screen. Participants inserted their right hands through a gap in the folding screen, which was obstructed by a curtain. To prevent movement, participants’ index fingers were secured to the table with double-sided tape. Filaments of between 0.41 and 0.06 mm – corresponding approximately to between 8.51 and 0.005 grams of force – were then applied to the participant’s index finger in four blocks (two ascending, two descending), with a dummy trial included in each block. Participants were asked to report whether or not they could feel the stimulus. In descending blocks, presentation was halted after two consecutive misses; in ascending blocks, presentation was halted after two consecutive hits. In the second block of each order, presentation began with filaments of the diameter from three trials before their previous threshold. If participants reported that they could feel the stimulus in a dummy trial, the block was repeated. Thresholds were calculated and reported using a log_10_ scale of approximate actual forces, corresponding to a linear scale of perceived forces, that was provided by the manufacturer (Stoelting Co, 2001). Approximate log forces from the final trials in each of the four blocks were averaged together to produce an estimate of the participant’s tactile detection threshold. Thresholds are reported here only from right-handed participants (i.e., those with positive laterality quotients on a modified Edinburgh Inventory; see Oldfield, 1971) because tactile thresholds could differ between dominant and non-dominant hands (Ghent, 1961). Measures from 31 autistic and 20 typically developing right-handed participants were available.

#### Tactile Spatial Resolution

JVP domes (named for Johnson, Van Boven, and Phillips; see Van Boven et al., 2000; Stoelting Co, 2021) were used to estimate tactile spatial resolution. JVP domes are hemispherical plastic domes, the heads of which contain gratings with equidistant bars and grooves. Different domes have different bar and groove widths which vary between 3.00 and 0.35 mm. Participants were seated in front of the opaque screen described above, in a procedure adapted from Bleyenheuft et al. (2006); JVP domes were applied to the index finger, with participants being instructed to report whether the orientation was “sideways” or “longways.” Participants completed five practice trials with the largest grating before the same grating was applied in random orientations for ten trials. The test then continued with ten further trials using the next-largest grating, and so on, until the probability of correct answers reached 50% at any grating level. Seventy-five percent tactile spatial acuity thresholds were estimated using linear interpolation (Van Boven and Johnson, 1994; Bleyenheuft et al., 2006):


$$\text{Threshold}=g_{below}+\frac{0.75-p_{below}}{p_{above}-p_{below}}\left(g_{above}-g_{below}\right)$$

Here, *g* is grating width and *p* is the probability of reporting the correct orientation at a given grating width. The grating width “above” means the lowest width yielding *p* greater than 75%, while the grating width “below” means the following grating width (where *p* has dropped below 75%). Where *p* remained below or above 75% at all grating widths tested, thresholds were recorded as 3.00 mm or 0.35 mm, respectively. Thresholds were obtained from 32 autistic and 19 typically developing right-handed participants.

### Questionnaire and Psychophysical Analyses

#### Autism Spectrum Development – Typical Development Group Comparisons

Comparisons of questionnaire scores in the auditory and tactile modalities, and tactile thresholds, between the ASD and TD groups used Wilcoxon–Mann–Whitney tests. Furthermore, when any ordinal tests reached statistical significance, linear parametric ANCOVA was used to explore whether covarying for WISC PRI scores would attenuate effects. Analyses of the questionnaire data were corrected using the Holm–Bonferroni procedure, separately for auditory and tactile subscales.

Comparisons of summed questionnaire factor scores, collapsing across all modalities, are presented in Supplementary Figure 2 and Supplementary Table 1.

For comparisons of hearing thresholds, we employed a two-tailed maximum-based permutation test (using a custom R script adapted from Gondan and Minakata, 2016). Cliff’s δ (Cliff, 1993), an ordinal effect size measure, was used to compare hearing thresholds between the ASD and TD groups at each frequency for each ear, separately. The maximum value of δ – the largest group difference in hearing thresholds – was then compared to a permutation distribution of 10,001 δ values. In addition, for ease of interpretation, we used Wilcoxon-Mann-Whitney tests to compare groups on PTAs, thresholds averaged across frequencies.

#### Psychophysical – Questionnaire Associations

Pearson’s correlation coefficient (*r*) was used to index associations between questionnaire scores and psychophysical auditory and tactile thresholds within the ASD group. Hearing thresholds were averaged across frequencies and ears.

We focused analyses on questionnaire subscales and items that one might expect to be the most relevant to psychophysical thresholds in the auditory and tactile modalities.

Ausderau et al. (2014) suggest that the SEQ-3.0’s EP pattern reflects “superior acuity in the awareness of specific sensory stimuli” and might be related to low thresholds. We therefore examined the correlation between auditory thresholds and auditory EP; due to the presence of only one tactile EP item, we did not explore correlations between tactile thresholds and SEQ scores.

As noted above, for the purpose of this correlation analysis, we summed sensory sensitivity and sensory avoiding items from the AASP to generate AASP “Low Threshold” scores for both the auditory and tactile modalities.

Finally, we examined correlations between psychophysical thresholds and the SP’s auditory and tactile “Low Threshold” items.

### Neurophysiological Measures

#### Electroencephalography Procedure and Stimuli

Electrophysiological data in this study were collected as part of a trimodal multisensory integration task, in which auditory, somatosensory, visual, audio-somatosensory, audiovisual, visuo-somatosensory, and audiovisual-somatosensory stimuli were intermixed at a random interstimulus interval of 1000–2250 ms. A total of 920 stimuli (∼130 per condition) were presented in ten blocks. Only data from the auditory-alone and somatosensory-alone conditions are included in this report.

**FIGURE 1:**
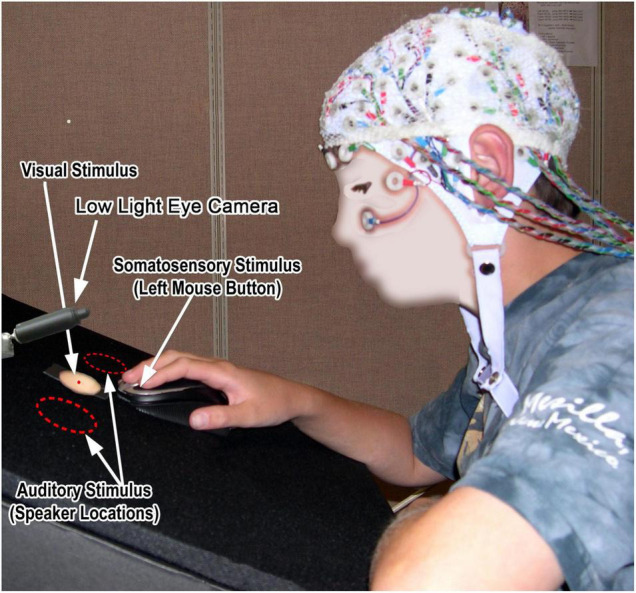
Twelve-year child pilot participant seated at the tri-sensory stimulation desktop.

Stimuli were delivered while participants were seated in a dimly-lit, electrically shielded, quiet testing chamber in front of a custom-built desktop stimulus delivery and RT-recording apparatus (Figure 1). Auditory stimuli were 20 ms, 63 dB SPL (at participants ears) noise bursts shaped to the average spectrum of human speech (Cox and Moore, 1988) in order to increase activation of lateral belt areas of the spatial auditory system (Maeder et al., 2001; Rauschecker and Tian, 2004). Sounds were delivered monophasically from two 3′′ loudspeakers (JBL gto326) located to either side of the central visual stimulus location and the resultant sound appeared to come from the same location as the locations of the visual and somatosensory stimuli.

Somatosensory stimuli were 8 ms mechanical taps (single cycles of a 120 Hz cosine wave) delivered to participants’ right index fingers as they placed their hand on an immovable mouse at the center of the desktop (Figure 1). Stimuli were delivered by actuating the left mouse button using a linear motor mounted below the mouse. To deliver what would be perceived as a single tap with no overshoot or rebound, the linear motor (a modified 3′′ Fosgate Punch car radio speaker) was driven by a low distortion audio signal using a Benchmark DAC1 digital-to-analog converter and Hafler Transnova amplifier whose extended low-frequency response and high damping factor eliminated perceptible overshoot and rebound. To ensure repeatable levels of tactile stimulation, finger pressure on the left mouse button was continuously measured using a Grass FT03 Force-Displacement Transducer located in line with the linear motor output (see Supplementary Figure 1). The output of the force transducer via a Grass P22 amplifier was fed into a Coulbourn V21-10 window discriminator, which was adjusted for each participant to define a pressure window representing light finger pressure on the mouse button. Too light, in-range, and excessive pressure were indicated via a light box visible to the experimenter with the participant and trials only proceeded with in-range pressure. Intensity of the tactile tap was adjusted to be subjectively roughly equivalent to the modest noise bursts and visual stimuli. The force transducer also served to generate a button-press motion signal used to determine RT.

Although careful alignment, acoustic shielding and robust construction of somatosensory stimulator materials mitigated acoustic output from the somatosensory stimuli, a quiet thump could nonetheless be heard accompanying the stimuli. This sound was effectively masked by continuously playing a low-frequency noise signal (peak power between 100 and 200 Hz) in the background during the experiment.

Visual stimuli (not reported here) were generated by 20 ms illumination of a strip of LEDs diffused using a translucent circular opening in the center of the desktop (see Figure 1). These flash stimuli were 85 cd/m^2^ with a 3:2 contrast ratio.

Participants were instructed to fixate a small red LED at the center of the circular opening and respond to *all* stimulus events (whether alone or in simultaneous combination) by pressing the left mouse button with their right index finger as quickly as possible. This button was also used to mechanically deliver the somatosensory stimuli to the right index finger. Fixation compliance was monitored using a low-light camera focused on the participants eyes. The experimenter in the recording chamber halted the delivery of stimuli when fixation was lost. Thus, electroencephalography (EEG) data were recorded as part of an active behavioral task, in a controlled environment, and only while participants appeared (overtly, via fixation, at least) to be attending to stimuli. This tightly controlled data acquisition context, with an active task requiring sustained attention, should certainly be borne in mind when considering convergence with other sensory measures.

Admittedly, the intermixed nature of the stimuli makes this a multisensory context, imposing a need to frequently switch attention between modalities, and there may be ASD-TD differences in multisensory attention switching (Occelli et al., 2013). However, the lab environment is still a strictly controlled one; there are no distracting background stimuli, and stimuli in all modalities emanate from the same multisensory desktop. Furthermore, it should be noted that the intermixed presentation of these stimuli might reduce habituation/repetition suppression and make habituation less of a contributor to any ASD-TD group differences.

#### Electroencephalography Acquisition and Processing

Continuous EEG was recorded from 125 Ag/AgCl scalp electrodes in an equidistant Montage^2^ and digitized at 1000 Hz (Compumedics Neuroscan Synamp2) with Cz as a reference. Three-dimensional electrode locations for each individual participant relative to bony fiducials were obtained using a Polhemus Patriot magnetic field-based 3D digitizer. Eye movements were monitored using horizontal and vertical electrooculography (EOG). Data were then imported into BESA Research 5.3, low-cut filtered (0.4 Hz, forward causal, 6 dB/oct roll-off), epoched (–200 ms to +1100 ms), and average-referenced. Trials with extreme amplitudes, trials with EOG events between –200 ms and +400 ms, and trials lacking behavioral responses were removed and bad channels were indicated. The remaining data were then entered into a second-order blind source identification (SOBI) independent components analysis using custom MATLAB code with advanced visualization capabilities provided by the Semi-Automatic Artifact Removal Tool (SMART; Saggar et al., 2012). Putatively neural components were then reconstructed with epochs spanning –200 to +800 ms. Averages were generated for the unisensory auditory and somatosensory conditions. The averaged data were exported to CARTOOL (Brunet et al., 2011) and inspected for electrolyte bridging and any further bad channels (heavily contaminated channels may be reconstructed with very low amplitude after removal of artifactual signal source). Bad channels were interpolated via three-dimensional spline (Perrin et al., 1987). ERPLAB (Lopez-Calderon and Luck, 2014) was used to apply high-cut filters (50 Hz Butterworth, zero-phase, 24 dB/oct) and to apply a baseline correction (100 ms prestimulus).

Usable ERP data were obtained from 33 autistic and 18 typically developing participants. Counts of usable ERP trials, and of trials eliminated during data processing, are presented by diagnostic group and modality condition in Table 2. Overall, autistic participants had significantly fewer usable somatosensory trials and trended toward having fewer auditory trials than typically developing participants.

**TABLE 2:** Total counts of retained and rejected trials in ERPs by diagnostic group and modality condition (A, auditory, S, somatosensory).

	Retained Trials						Rejected Trials					
	ASD		TD		*p*	δ	ASD		TD		*p*	δ
	Mean (*SD*)	Range	Mean (SD)	Range			Mean (*SD*)	Range	Mean (*SD*)	Mean (SD)		
A	87.67 (21.89)	48–132	98.94 (21.74)	49–134	0.08	−0.30	42.00 (21.49)	5–81	32.61 (20.91)	6–76	0.15	0.25
T	86.30 (21.55)	53–128	99.44 (15.49)	71–124	0.02	−0.39	42.97 (22.90)	10–82	31.61 (17.18)	10–69	0.08	0.30

*Mean counts and standard deviations are given, the latter in brackets, along with ranges. Wilcoxon–Mann–Whitney rank-sum tests are used to compare totals across groups; δ (Cliff, 1993) is reported as an effect size.*

#### Event-Related Potentials Analysis

We chose to focus ERP analyses on the canonical auditory Tb and somatosensory P60 components, due to their developmental stability in the age range of the present study (see Albrecht et al., 2000; Ponton et al., 2002; Uppal et al., 2016) and their prominence over the scalp.

**FIGURE 2:**
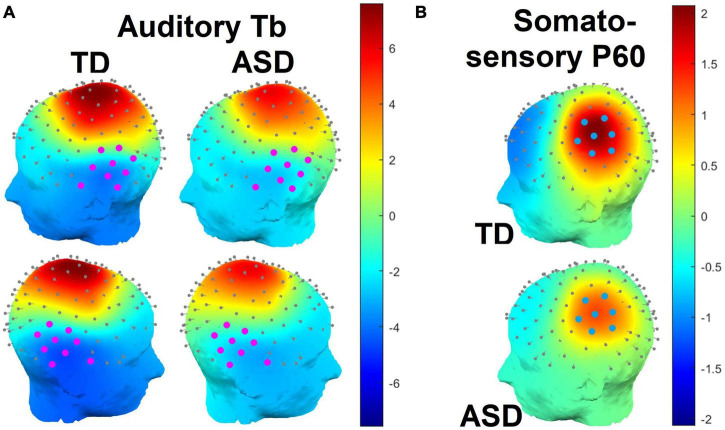
**(A)** Splined scalp topographies over the left and right hemispheres in the time window of the auditory Tb response (135–195 ms). Electrodes used to the measure the Tb response are highlighted in magenta. Note that electrode positions are based on observations from a Polhemus digitizer and may appear slightly irregular as a result. **(B)** Splined scalp topographies over the left (contralateral) hemisphere in the time window of the somatosensory P60 response (38 – 78 ms). Electrodes used to the measure the P60 response are highlighted in blue.

**FIGURE 3:**
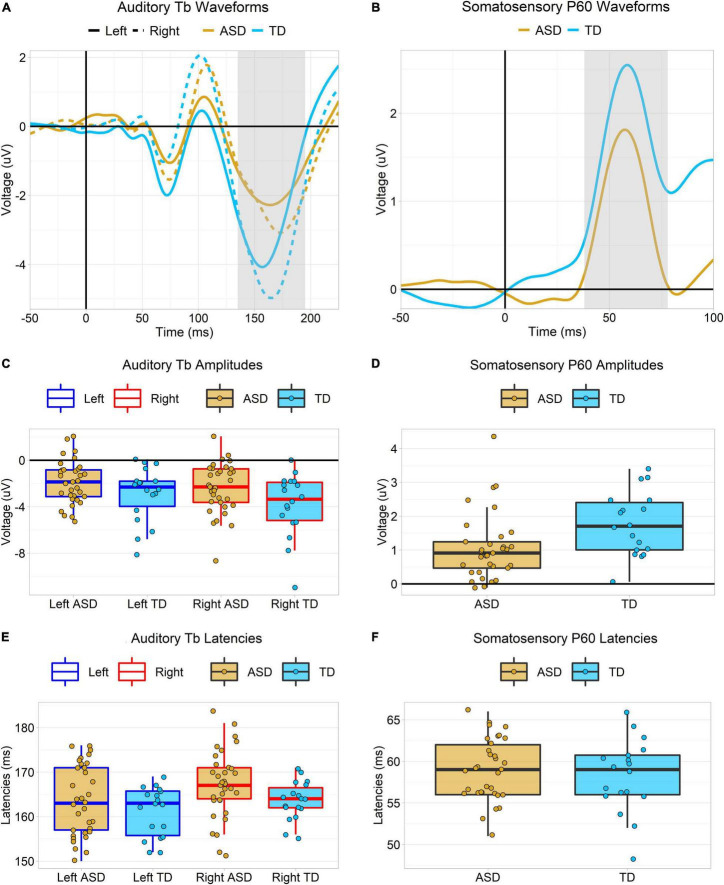
Plots depicting Auditory Tb and somatosensory P60 ERP responses. **(A,B)** Tb and P60 ERP waveforms in each group. P60 responses are contralateral (left hemisphere only), but the Tb is shown over each hemisphere. The gray highlighted regions represent the measurement windows (Tb: 135–195 ms, P60: 38–78 ms). **(C,D)** Boxplots showing amplitudes of the Tb and P60 ERP responses. Amplitudes of both were greater in TD then ASD, but these effects dropped below the threshold for statistical significance after covarying for WISC PRI scores. Tb amplitudes were larger over the left hemisphere than the right. **(E,F)** Boxplots showing latencies of the Tb and P60 ERP responses. Latencies of the auditory Tb were faster in TD than ASD, but this effect was no longer significant after covarying for WISC PRI scores. Tb latencies were faster over the left hemisphere than the right.

The auditory Tb response was examined by averaging across a set of left and right hemisphere temporal channels selected by visual inspection to align with the observed scalp topography of the grand average Tb (see Figure 2A below) collapsed across groups. The Tb time window was defined as ±30 ms on either side of the grand-averaged Tb peak across both hemispheres and groups (165 ms), or 135–195 ms (Figure 3A). Tb amplitudes over each hemisphere were the mean amplitudes across electrodes over this window. Tb latencies were 50% fractional area latencies within this window (i.e., the time points that divided the waveform area into two equal halves; see Luck, 2014, pp. 296–299).

The somatosensory P60 response was examined over contralateral (left hemisphere) centro-parietal channels that, by visual inspection, appeared to correspond to the observed scalp topography of the P60 (see Figure 2B). Due to the narrowness and sharpness of this early component, the P60 time window was defined as ±20 ms on either side of the grand-averaged P60 peak across both groups (58 ms), or 38–78 ms (Figure 3B). P60 amplitudes were quantified as mean amplitudes and P60 latencies as 50% fractional area latencies.

##### Autism Spectrum Development – Typical Development Group Comparisons

ANOVA was used to compare ERP amplitudes and latencies across groups and, in the case of the Tb response, across hemispheres. Significant group differences were subsequently probed with ANCOVA, covarying for WISC PRI scores, the number of retained ERP trials, and sensory thresholds.

##### Event-Related Potentials – Psychophysical/Questionnaire Associations

Pearson’s correlation coefficient (*r*) was used to index associations, within the ASD group, between ERP amplitudes and psychophysical thresholds as well as questionnaire scores within the same modality. For these analyses, auditory Tb amplitudes were averaged across hemispheres and pure tone average hearing thresholds were collapsed across ears.

ERP-psychophysical correlations were corrected for three multiple comparisons using the Holm-Bonferroni procedure. Correlations between the auditory Tb and somatosensory P60 and questionnaire subscales were corrected for ten and nine comparisons, respectively, using the Holm-Bonferroni procedure; the discrepancy is due to the absence of an SEQ tactile EP subscale.

## Results

### Psychophysical and Questionnaire Group Comparisons

#### Adolescent/Adult Sensory Profile

Overall, autistic participants appeared to have higher AASP low registration, sensory sensitivity, and sensation avoiding scores than typically developing participants in both the auditory (Table 3) and tactile (Table 4) modalities. However, when multiple comparison corrections were applied and after covarying for cognitive ability, trending differences in auditory sensory sensitivity and tactile sensation avoiding were no longer significant.

**TABLE 3:** Results of statistical comparisons of autistic and typically developing groups on Sensory Profile (SP), Adolescent-Adult Sensory Profile (AASP), and Sensory Experiences Questionnaire (SEQ) scores in the auditory modality.

	Mean (*SD*)		Wilcoxon–Mann–Whitney Effect			ANCOVA Effect		
	ASD	TD	Cliff’s δ	*p*	Corrected *p*	*F*	*p*	Corrected *p*
SP Auditory Low Threshold	15.12 (3.88)	23.00 (2.71)	–0.87	<0.0001	<0.0001	46.34	<0.0001	<0.0001
SP Auditory High Threshold	8.97 (2.48)	12.84 (1.92)	–0.77	<0.0001	<0.0001	39.75	<0.0001	<0.0001
AASP Auditory Low Registration	8.90 (2.97)	5.00 (1.78)	0.72	<0.0001	<0.0001	18.37	<0.0001	0.0003
AASP Auditory Sensory Sensitivity	8.74 (3.13)	6.22 (3.15)	0.46	0.007	0.01	4.32	0.04	0.09
AASP Auditory Sensation Avoiding	8.91 (3.18)	5.83 (2.15)	0.55	0.001	0.004	7.45	0.009	0.03
AASP Auditory Sensation Seeking	6.27 (2.33)	6.67 (2.11)	–0.12	0.46	>0.46	0.20	0.66	0.66
SEQ Auditory HYPO	6.30 (2.09)	3.95 (1.07)	0.70	<0.0001	<0.0001	21.06	<0.0001	0.0001
SEQ Auditory HYPER	11.34 (3.77)	4.43 (1.17)	0.96	<0.0001	<0.0001	54.93	<0.0001	<0.0001
SEQ Auditory SIRS	4.61 (2.14)	2.33 (0.73)	0.66	<0.0001	<0.0001	19.29	<0.0001	0.0002
SEQ Auditory EP	10.02 (3.94)	4.95 (0.97)	0.84	<0.0001	<0.0001	28.96	<0.0001	<0.0001

*Means and standard deviations in each group are presented on the left. Results of ordinal Wilcoxon–Mann–Whitney tests are presented on the center, along with associated Cliff’s δ effect sizes. Lower scores on SP and higher scores on AASP and SEQ indicate greater levels of sensory patterns on each subscale. Thus, negative δ for SP and positive δ for AASP and SEQ indicate greater levels of sensory patterns in the ASD group than the TD group. Results of ANCOVA analyses covarying for WISC PRI scores are presented on the right. Corrected p-values employ the Holm–Bonferroni procedure to correct for 10 comparisons, separately for ordinal and ANCOVA analyses.*

**TABLE 4:** Results of statistical comparisons of autistic and typically developing groups on Sensory Profile (SP), Adolescent-Adult Sensory Profile (AASP), and Sensory Experiences Questionnaire (SEQ) scores in the tactile modality.

	Mean (*SD*)		Wilcoxon–Mann–Whitney Effect			ANCOVA Effect		
	ASD	TD	Cliff’s δ	*p*	Corrected *p*	*F*	*p*	Corrected *p*
SP Tactile Low Threshold	38.32 (9.19)	51.64 (4.80)	–0.82	<0.0001	<0.0001	27.92	<0.0001	<0.0001
SP Tactile High Threshold	26.50 (5.21)	32.11 (2.90)	–0.64	0.0001	0.0006	14.07	0.0005	0.003
AASP Tactile Low Registration	7.53 (2.61)	5.67 (1.94)	0.42	0.01	0.04	7.75	0.008	0.02
AASP Tactile Sensory Sensitivity	10.20 (3.03)	7.33 (2.95)	0.51	0.003	0.01	9.64	0.003	0.01
AASP Tactile Sensation Avoiding	7.79 (3.10)	5.72 (2.27)	0.41	0.02	0.03	3.41	0.07	0.14
AASP Tactile Sensation Seeking	7.79 (2.83)	6.83 (3.28)	0.20	0.23	0.23	0.34	0.56	0.56
SEQ Tactile HYPO	8.45 (3.92)	4.81 (1.17)	0.63	<0.0001	0.0002	12.69	0.0007	0.004
SEQ Tactile HYPER	29.19 (9.73)	15.76 (5.43)	0.79	<0.0001	<0.0001	28.98	<0.0001	<0.0001
SEQ Tactile SIRS	17.06 (5.04)	8.33 (1.91)	0.93	<0.0001	<0.0001	53.21	<0.0001	<0.0001

*Means and standard deviations in each group are presented on the left. Results of ordinal Wilcoxon–Mann–Whitney tests are presented on the center, along with associated Cliff’s δ effect sizes. Lower scores on SP and higher scores on AASP and SEQ indicate greater levels of sensory patterns on each subscale. Thus, negative δ for SP and positive δ for AASP and SEQ indicate greater levels of sensory patterns in the ASD group than the TD group. Results of ANCOVA analyses covarying for WISC PRI scores are presented on the right. Corrected p-values employ the Holm–Bonferroni procedure to correct for nine comparisons, separately for ordinal and ANCOVA analyses.*

Levels of AASP sensation seeking did not even trend toward differing between autistic and typically developing participants, even before multiple comparison corrections were applied, in either the auditory or tactile modalities.

#### Sensory Experiences Questionnaire-3.0

Autistic participants had significantly and robustly higher SIRS, HYPO, and HYPER scores than typically-developing participants in both the auditory (Table 3) and tactile modalities (Table 4); these high scores reflect higher levels of each of these sensory patterns. Furthermore, autistic participants had higher auditory EP scores.

As noted above, tactile EP was not examined due to reliance on a single item.

#### Sensory Profile

Autistic participants had significantly greater “low threshold” and “high threshold” scores on the SP in both the auditory (Table 3) and tactile modalities (Table 4).

#### Hearing Acuity

**FIGURE 4:**
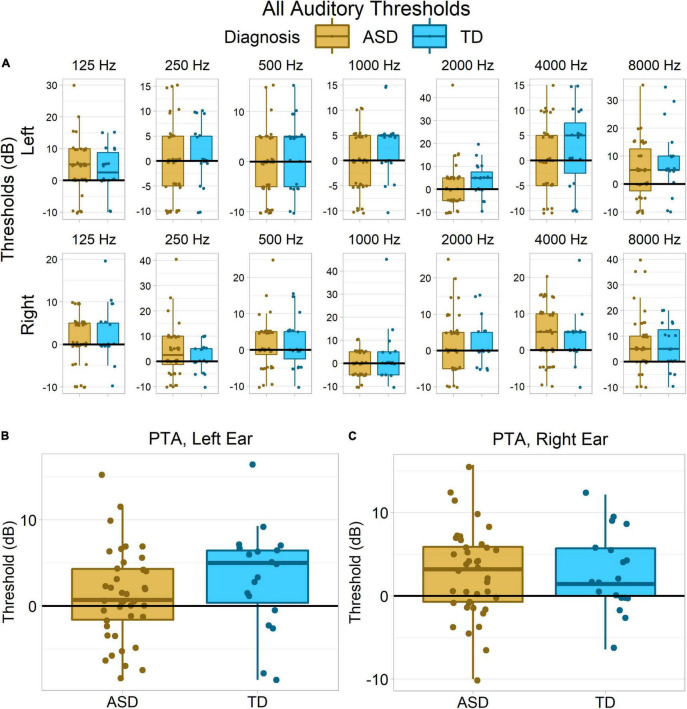
Hearing acuity in each group. No statistically reliable group differences were observed, despite the apparent trend toward greater auditory acuity in the ASD group in the left ear **(B)**. **(A)** Hearing thresholds for each tone frequency and ear separately. **(B,C)** Pure tone average thresholds (collapsing across frequencies) for each ear.

Permutation testing across both ears and all frequencies found a modest trend toward between-group differences in hearing acuity, *p* = 0.09 (Figure 4A). Groups did not significantly differ in pure tone average (PTA) from right ear, Wilcoxon–Mann–Whitney *p* = 0.94, δ = −0.01 (Figure 4C), or collapsing across both ears, *p* = 0.25, δ = −0.19. However, there was a small and non-significant trend for the autistic group to have lower PTA thresholds – better hearing acuity – in the left ear, *p* = 0.11, δ = −0.26 (Figure 4B).

#### Tactile Static Detection

**FIGURE 5:**
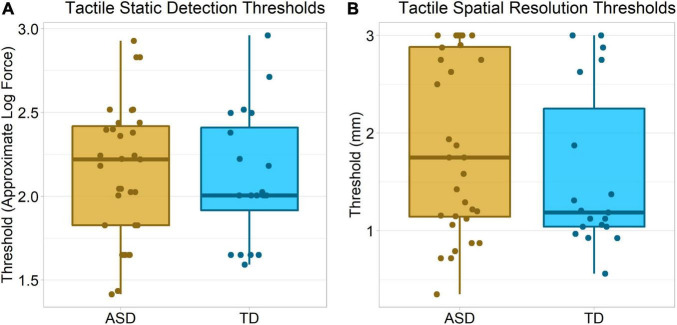
Tactile thresholds in each group. **(A)** Thresholds for detection of static (non-vibrating) tactile stimuli, specifically Von Frey hairs, using an approximate/theoretical log scale of forces provided by the manufacturer (Stoelting Co, 2001). **(B)** Thresholds for recognition of the orientations of tactile gratings of different widths (in mm).

There was no significant group difference in Von Frey hair detection thresholds, Wilcoxon–Mann–Whitney *p* = 0.50, δ = 0.11 (Figure 5A).

#### Tactile Spatial Resolution

There was no significant group difference in tactile spatial acuity, Wilcoxon–Mann–Whitney *p* = 0.21, δ = 0.21. Interestingly, visual inspection of the data (Figure 5B) suggests a bimodal distribution of participants, with one group of participants having higher thresholds. While this might in part reflect a floor/ceiling effect, prior research suggests thresholds around 1 – 2 mm, within the range of our equipment, are typical in this age range (Bleyenheuft et al., 2006, 2010; Peters and Goldreich, 2013).

### Psychophysical – Questionnaire Associations

After correcting for multiple comparisons, we found no significant correlations between hearing thresholds and AASP/SP auditory low threshold scores or SEQ auditory EP scores; between Von Frey thresholds and AASP/SP Tactile Low Threshold scores; or between JVP dome thresholds and AASP/SP Tactile Low Threshold scores, all Holm–Bonferroni corrected *p’s* ≥ 0.92 (Supplementary Table 2).

### Event-Related Potentials Group Comparisons

#### Auditory Tb

In a mixed ANOVA, the amplitude of the auditory Tb response was larger in typically developing than autistic participants, *F*(1,49) = 4.47, *p* = 0.04, $\eta_{G}^{2}$ = 0.08 (Figures 2A, 3A,C). Using ANCOVA to covary for WISC PRI scores and the number of retained trials slightly attenuated the main effect of diagnostic group such that it was no longer significant, *F*(1,47) = 3.28, *p* = 0.08, and further covarying for hearing thresholds (in those participants with hearing threshold data) attenuated it more, *F*(1,44) = 1.56, *p* = 0.22. Amplitudes were also larger over the right hemisphere than the left, *F*(1,49) = 6.06, *p* = 0.02, $\eta_{G}^{2}$ = 0.02. There was no interaction of group and hemisphere, *F*(1,49) = 0.64, *p* = 0.43, $\eta {}_{G}^{2}$ = 0.00.

Tb latencies were slightly faster in TD participants than ASD participants, *F*(1,49) = 4.17, *p* < 0.05, $\eta_{G}^{2}$ = 0.04 (Figures 3A,E). However, using ANCOVA to covary for WISC PRI scores and the number of retained trials attenuated the main effect of diagnostic group such that it no longer approached significance, *F*(1,47) = 1.98, *p* = 0.17, though further covarying for hearing thresholds decreased the *p*-value slightly, *F*(1,44) = 2.93, *p* = 0.09. Tb latencies were slightly faster over the left hemisphere than the right, *F*(1,49) = 4.89, *p* = 0.03, $\eta_{G}^{2}$ = 0.04. There was no interaction of group and hemisphere, *F*(1,49) = 0.22, *p* = 0.64, $\eta {}_{G}^{2}$ = 0.00.

#### Somatosensory P60

The amplitude of the somatosensory contralateral (left hemisphere) P60 response was larger in the typically developing group than the autistic group, *F*(1,49) = 6.19, *p* = 0.02, $\eta_{G}^{2}$ = 0.11 (Figures 2B, 3B,D). However, using ANCOVA to covary for WISC PRI scores and the number of retained trials attenuated this main effect of diagnostic group such that it was no longer significant, *F*(1,47) = 2.91, *p* = 0.09. Covarying for tactile static detection thresholds in participants with those data had little further effect, *F*(1,43) = 2.62, *p* = 0.11.

There was no effect of diagnostic group on the latency of the P60, *F*(1,49) = 0.41, *p* = 0.52, $\eta_{G}^{2}$ = 0.01 (Figures 3B,F).

### Event-Related Potential – Psychophysical Correlations

#### Auditory Tb

**FIGURE 6:**
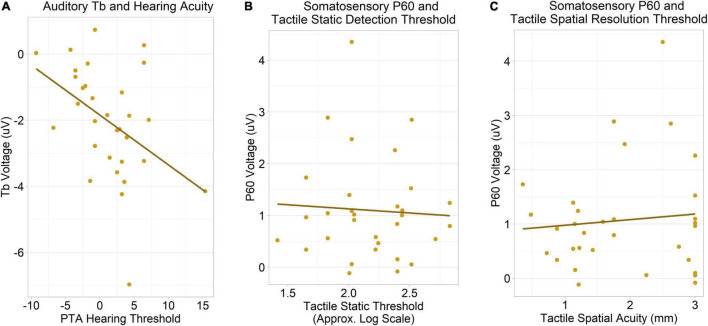
**(A)** A negative correlation was observed between auditory Tb amplitudes (averaged across hemispheres) and pure tone average hearing thresholds (averaged across ears), corrected *p* = 0.04. Despite the apparent outliers, the corrected *p*-value remained significant when the association was measured ordinally (using Kendall’s τ). **(B)** No association between contralateral somatosensory P60 amplitudes and tactile static detection (Von Frey hair) thresholds was observed. **(C)** No association between somatosensory P60 amplitudes and tactile spatial resolution (JVP dome) thresholds was observed.

Unexpectedly, in the ASD group, we observed a significant negative correlation between auditory Tb amplitudes and pure tone average hearing thresholds collapsed across both ears, *r*(29) = −0.43, Holm–Bonferroni corrected *p* = .04 (Figure 6A): that is, autistic individuals with *larger* (more negative) Tb responses had reduced hearing acuity/higher thresholds. This association remained robust when measured ordinally, Kendall’s τ = −0.34, uncorrected *p* = 0.008, suggesting it is not driven by the visually apparent outliers in Figure 6A.

Due to the unexpected nature of this effect, we carried out an unplanned analysis to explore whether motor confounds could have accounted for it. However, after removal of an outlier, we found no correlation between Tb amplitudes and participants’ median reaction times to auditory stimuli in the ERP task, *r*(30) = −0.15, *p* = 0.42 (ordinally, with outlier present, τ = −0.18, *p* = 0.14).

#### Somatosensory P60

There was no association between contralateral P60 amplitudes and tactile static detection (Von Frey hair) thresholds, *r*(29) = −0.06, uncorrected *p* = 0.76 (Figure 6B), nor was there an association between P60 amplitudes and tactile spatial resolution thresholds, *r*(30) = 0.09, uncorrected *p* = 0.61 (Figure 6C).

### Event-Related Potential – Questionnaire Correlations

We found no significant correlations between auditory Tb amplitudes and the ten different auditory subscale scores from the AASP, SP, and SEQ, all Holm-Bonferroni corrected *p* ≥ 0.52 (Supplementary Table 3), or between somatosensory P60 amplitudes and the nine different tactile factors from the AASP, SP, and SEQ, all Holm–Bonferroni corrected *p* ≥ 0.14 (Supplementary Table 4).

## Discussion

The present study examined sensory processing in young autistic adolescents, as well as a TD comparison group, using multiple measurement modalities: questionnaires, psychophysical thresholds, and ERPs. Briefly, we found autistic participants were reported to display more atypical and intense sensory behaviors across almost all questionnaire subscales, either significantly or at a trend level; however, there was much more limited evidence of group differences in psychophysical thresholds. While there was a trending group difference in hearing acuity, apparently driven by the left ear, there were no group differences in tactile static detection or tactile spatial resolution thresholds. Autistic participants initially appeared to have slower auditory Tb latencies and reduced Tb and somatosensory P60 amplitudes, although these effects were no longer significant after covarying for WISC PRI cognitive scores, numbers of retained ERP trials, and psychophysical thresholds in each modality.

The present study also examined convergence among these measures in the ASD group. We found no associations between questionnaire scores and either psychophysical thresholds or ERP amplitudes. However, we were surprised to observe a significant negative correlation between auditory Tb amplitudes and hearing thresholds, such that participants with reduced hearing acuity displayed larger neural responses to the noise bursts presented in our ERP task.

### Questionnaires

Our overall finding that the autistic participants reportedly displayed more atypical and intense sensory behaviour on most questionnaire subscales/factors in the auditory and tactile modalities, compared to typically developing participants, is quite consistent with prior research (e.g., Baranek et al., 2006; Kern et al., 2006; Weiland et al., 2020) and with Hypothesis 1. This finding also emphasizes the extent and importance of atypical autistic sensory behaviors in participants’ real-world environments.

Of nineteen subscales/factors in the auditory and tactile modalities, fifteen yielded statistically reliable group differences, two yielded trends that ceased to be significant after correction for multiple comparisons and after covarying for cognitive ability, and only two showed no trend at all.

Only the AASP Sensation Seeking displayed, in both the auditory and tactile modalities, no hint of discriminating between autistic and typically-developing participants even at a trend level. However, autistic participants displayed more Sensory Interests/Repetitions/Seeking (SIRS) on the SEQ. Furthermore, supplementary analyses examining total AASP Sensation Seeking scores (collapsing across sensory modalities) found no group differences in AASP Sensation Seeking, despite the presence of robust group differences in SEQ SIRS and SP Sensation Seeking (Supplementary Figure 2 and Supplementary Table 1).

Interestingly, in some prior studies using the AASP, autistic participants have even reported engaging in *less* sensation seeking behavior than typically developing individuals (Crane et al., 2009; Mayer, 2017). Elwin et al. (2013) explain these results by noting that sensation seeking items on the AASP, which were developed for the general population (e.g., “I touch others when I’m talking,” “I like to attend events with a lot of music”), may not capture the sorts of sensation seeking behaviors preferred by many autistic people. This appears to be the most plausible explanation for the present study’s lack of group differences in AASP Sensation Seeking. As the AASP is not equivalent to the SP or SEQ, and as prior research supports the validity of self-report sensory measures in ASD (Keith et al., 2019), the fact the AASP was completed by participants themselves rather than by their caregivers seems unlikely to have been responsible.

Overall, it is striking that many of the largest ASD-TD group differences, as indexed by the Cliff’s δ effect size metric (see Tables 3, 4 and Supplementary Table 1), were observed on the SEQ — a measure that was specifically developed with autism in mind (Baranek et al., 2006; Ausderau et al., 2014). This appears to encourage the use of sensory measures developed for autistic populations in assessments of autistic people’s sensory processing.

### Psychophysical Measures

As predicted by Hypothesis 2, the ASD and TD groups did not significantly differ in any of the three psychophysical threshold measures collected in the present study. Our failure to find clear and consistent ASD-TD differences in hearing thresholds appears consonant with prior literature (Khalfa et al., 2004; Gravel et al., 2006; Demopoulos and Lewine, 2016; Kuiper et al., 2019). Furthermore, the present study’s failure to find ASD-TD differences in thresholds for the perception of Von Frey hairs is also consistent with prior research (Cascio et al., 2008; Fukuyama et al., 2017).

Event-related potentials such as the mismatch negativity (Näätänen and Kreegipuu, 2011; Schwartz et al., 2018) can also be used to investigate discrimination between stimuli. While the large number of trials needed to generate stable ERP averages, combined with the potential for confounding factors such as skull thickness (Pfefferbaum, 1990) to affect ERPs, might make ERPs a generally less promising measure of stimulus discrimination than psychophysical tasks, ERPs might be more suitable for younger or non-speaking individuals. However, prior literature has found only modest ASD-TD differences in auditory mismatch negativity amplitudes to non-speech stimuli (reviewed by Schwartz et al., 2018). Although this finding collapses across variations in study stimuli, modest ASD-TD differences appear suggestive of largely intact stimulus discrimination, which seems in line with the present study’s null results.

While these null results could suggest that the rigorously controlled environments of lab-based tasks – in which participants are often given explicit task instructions, and in which potentially competing or distracting sensory stimuli are often minimized – can sometimes be poorly suited toward investigating real-world autistic sensory experiences, there are ways of designing lab-based tasks to increase their naturalistic validity and/or relevance to autism. For example, if autistic hyper-sensitivities reflect stimulus discomfort rather than altered psychophysical thresholds, measures of stimulus discomfort, such as Loudness Discomfort Levels (see Khalfa et al., 2004), might yield more robust group differences than measures of stimulus detection and discrimination. These sorts of group differences might be most naturalistically valid if everyday, real-world sounds are included in task batteries (as in, e.g., Enzler et al., 2021a,b; Hansen et al., 2021). To further enhance naturalistic validity, paradigms could include distracting, task-irrelevant stimuli (e.g., Karhson and Golob, 2016; Keehn et al., 2016; Blomberg et al., 2021). Ideally, such paradigms would allow for controlled analyses of the effects of specific manipulations on sensory processing, without compromising naturalistic validity.

However, although we did not observe significant group differences in psychophysical thresholds in the present study, we did find a strong trend toward group differences in hearing acuity when we used a permutation test to search for effects across both ears and all frequencies. Examination of PTA thresholds from each ear suggests this effect was driven by non-significantly lower thresholds – i.e., higher acuity – in left ear in the ASD group.

Some caution should be exercised in the interpretation of this result. As noted earlier, the effect did not attain statistical significance. It is also unclear whether it reflects genuine differences in hearing acuity, as opposed to factors such as fatigue or attention to task, which might be particularly important in child samples. The left ear was always tested first, before the right ear, so it is possible that some autistic participants were initially slightly more attentive to the task but that this attention difference had lapsed by the second half of the testing session.

#### Psychophysical Correlations With Questionnaires

Moreover, in the ASD group, psychophysical thresholds were not related to questionnaire scores representing “low thresholds” or enhanced perception. This result suggests that any potential ASD-TD psychophysical threshold differences are unlikely to account for the sensory challenges experienced by many autistic people in their daily lives. It also appears consistent with prior research reporting that, in the general population, auditory thresholds are unrelated to questionnaire reports of auditory sensitivity (Schulz and Stevenson, 2021).

### Event-Related Potentials

As predicted by Hypothesis 3, we initially observed reduced ERP amplitudes and delayed ERP latencies in ASD relative to TD. Specifically, the amplitudes of the auditory Tb and somatosensory P60 responses appeared reduced in ASD and the latency of the auditory Tb was delayed. These ERP group differences were attenuated above thresholds for statistical significance when we covaried for WISC PRI cognitive scores, numbers of ERP trials, and sensory thresholds. Group differences in Tb amplitudes no longer approached significance; however, group differences in Tb latencies and P60 amplitudes remained as non-significant trends. It should be noted that much of the variance associated with WISC PRI scores was also associated with diagnostic group (see Table 1), which makes it difficult to interpret the results of this ANCOVA. Therefore, while we cannot definitively state that observed differences in somatosensory P60 amplitudes and auditory Tb latencies are driven by diagnostic group, we believe these data are suggestive of group differences, which would be consistent with prior literature (Russo et al., 2010; Marco et al., 2012; Williams et al., 2021a).

A number of potential mechanisms could account for group differences in somatosensory P60 amplitudes. Although some theories suggest autistic sensory processing is characterized by increased noise (Haigh, 2018), we believe the present study’s use of mean amplitude measures should make results robust to inter-trial amplitude variability relative to if peak-based measures had been used (Luck, 2014), although intra-trial latency variability could potentially influence results if some responses were partly outside our measurement window. Gaetz et al. (2017) do speculate that attenuated early somatosensory responses in ASD might reflect reduced GABAergic signaling, which is an intriguing possibility in light of research regarding tactile processing and excitatory and inhibitory neurotransmission in autism (Sapey-Triomphe et al., 2019; Wood et al., 2021). That said, it is not clear that autistic and typically-developing individuals differ in levels of GABA or glutamate (Kolodny et al., 2020; Wood et al., 2021). The possibility that heterogeneity within autism could contribute to results must also be borne in mind: it is possible that these ERP results might be driven by subgroups and/or they might reflect different processes in different autistic individuals. As for Tb latency, prior research suggests that variance in auditory M50 latencies in ASD is not tightly coupled with levels of white matter (Roberts et al., 2013, 2020) but exploratory findings suggest it might be related to GABA levels in some subgroups (Roberts et al., 2020). However, it is unclear whether these conclusions generalize to the Tb response. Ultimately, further research is required to draw definitive conclusions regarding mechanisms for these amplitude and latency differences.

We found no evidence of ASD-TD group differences in somatosensory P60 latencies, consistent with prior research (Marco et al., 2012).

#### Event-Related Potential Correlations With Questionnaires

We observed no significant associations between Tb or P60 amplitudes and sensory questionnaire scores from the respective sensory modalities of each ERP response (i.e., auditory and tactile). This might imply that the subjective experience of sensory stimuli in daily life in autism is not substantially related to neurophysiological event-related responses to stimuli delivered in a controlled laboratory environment when participants are given clear attentional and motor instructions and in the absence of distracting or overwhelming stimulation.

Phenomenologically, we believe this makes sense. When participants (or proxy reporters such as parents) fill out questionnaires such as the AASP and SEQ, they are reporting on sensory experiences and behaviors from their daily lives. Everyday environments often feature many changing stimuli at a single moment in time (e.g., other people moving, flickering lights, the wind outside); furthermore, people in these environments might have many matters occupying their attention, such as schoolwork, social relationships, and more. Different people might allocate their attention differentially across all of these sensory stimuli and internal cognitions and ruminations, and in particular, some people might have their attention captured (for better or worse) by particular sensory stimuli. Thus, some people might be (as per one SEQ item) bothered by everyday sounds, like a dishwasher or blender, while others might more easily ignore these sounds to focus on other things.

In contrast, in the ERP paradigm used in the present study, extraneous sources of sensory input were intentionally controlled wherever possible, minimizing the dangers of distraction and attention capture. Furthermore, participants were given an explicit task requiring them to allocate their attention toward the specific sensory stimuli of interest, not any other target.

While prior studies such as those of Dwyer et al. (2020) and Schwartz et al. (2020b) have found relationships between auditory ERPs and sensory questionnaire scores, both Dwyer et al. (2020) and Schwartz et al. (2020b) presented auditory stimuli while participants watched quiet or silent videos of personal interest. Thus, it seems possible that neurophysiological responses to sensory stimuli might track better with the daily sensory experiences of autistic individuals if recorded in an experimental paradigm with multiple stimuli competing for attention, more closely resembling the complex real-world environments on which sensory questionnaire reporting may be based. However, this does not explain some prior findings of psychophysical-questionnaire associations under more controlled conditions (e.g., Cascio et al., 2015; Karhson and Golob, 2016).

#### Event-Related Potential Correlations With Psychophysical Thresholds

As predicted by Hypothesis 4, somatosensory P60 amplitudes were not related to tactile thresholds. Surprisingly, however, we found a robust correlation between auditory Tb amplitudes and hearing thresholds. Not only was the mere existence of this association contrary to the present study’s Hypothesis 4, but the direction of the association suggests that individuals with *larger* Tb responses had *reduced* hearing acuity/higher thresholds.

A number of prior studies investigating links between auditory ERP amplitudes and hearing thresholds have done so in populations with hearing loss, and these studies have generally and unsurprisingly found reduced to absent auditory ERP, including oddball ERP, responses in individuals with high hearing thresholds (Pollock and Schneider, 1992; Hoth, 1993; Lightfoot, 2016). In contrast, other studies show enhanced neural responses, potentially attributable to compensatory processing, in individuals with clinically elevated hearing thresholds (Campbell and Sharma, 2013; Goossens et al., 2019). However, the present study was conducted in individuals with hearing thresholds within normal limits, and it is thus unclear whether or not similar compensatory processes can be expected. Fjell and Walhovd (2003) did examine auditory N1 responses to auditory tones in three groups of adults, including two groups with non-clinical thresholds (0–5 dB and 10–15 dB). Reported N1 amplitudes appeared slightly greater in the 10–15 dB threshold group, but this effect did not attain significance. In any case, latencies of the auditory Tb and frontocentral responses do not appear to covary (e.g., Ponton et al., 2002), and prior research finds more robust ASD-TD group differences in amplitudes and latencies of the Tb than the frontocentral N1 (Williams et al., 2021a), suggesting that measures of each of these two ERP components reflect fairly independent variation from the other component. Thus, it is unclear how these frontocentral N1 findings might relate to the Tb.

An alternative explanation for the larger Tb responses observed in autistic participants with higher hearing thresholds from the present study is that these might have reflected the influence of the RT task. Participants were instructed to respond rapidly to all events (without discrimination), and it is possible that participants with lower thresholds were able to more quickly accumulate sufficient evidence to initiate a motor response, whereas participants with higher thresholds might have had to build and maintain representations of sensory events over a longer period of time. Unfortunately, owing to the developmental transition in morphologies of earlier vertex auditory ERPs observable in participants in this age range (such that the auditory P1 of some participants has similar timing and topography to the central N1 of others), it was not possible to evaluate whether similar effects could be observed at these latencies.

Given the lack of convergence between questionnaire scores and either ERP amplitudes or psychophysical thresholds, it seems unlikely that neural hyper-responsiveness to sound in autistic people with high hearing thresholds accounts for many of autistic people’s real-world sensory challenges. However, this neural hyper-responsiveness could still have important methodological implications. Simply ensuring that all participants have clinically normal hearing levels may not be sufficient to prevent differences in hearing thresholds from confounding results of between-group auditory ERP studies. In the present study, when we covaried for both auditory thresholds and WISC PRI scores in our ERP analyses, a group difference we had previously observed in auditory Tb amplitudes largely disappeared.

### Limitations

Although the present study has a number of strengths, including the large number of tools in different measurement modalities (questionnaire, neurophysiological, and psychophysical) used to explore sensory processing, the relatively narrow age range of the participants, and the well-characterized nature of the sample, the study is not without limitations.

One notable limitation is that the autistic and typically developing samples are most likely not from otherwise equivalent populations. There are large ASD-TD differences in cognitive ability (see Table 1), with typically developing participants often showing unexpectedly high scores relative to population norms. This might be because autistic participants were recruited from a broad geographical region, whereas typically developing participants were largely recruited from the immediate environs of Davis, CA, United States. Davis is an unrepresentative community, with unusually high educational attainments (United States Census Bureau, 2019). We did not collect information regarding socioeconomic status or parental education levels and are therefore unable to determine whether and how these factors might have influenced group comparisons. Furthermore, although we attempted to use ANCOVA to determine whether significant group differences might be explained by cognitive abilities, we acknowledge that this procedure has limitations; it is difficult in the present sample to separate variance associated with cognitive ability from variance associated with diagnosis. Fortunately, we believe the main contribution of the present study consists in its examination of convergence between different types of measures in ASD, rather than ASD-TD group differences in specific measures, making this limitation of arguably secondary importance.

We also chose to focus our analyses on the auditory Tb and the somatosensory P60. These were selected to minimize multiple comparisons and because they were the first large and developmentally stable cortical responses clearly visible in ERP averages (the morphology of earlier auditory responses changes dramatically in this age range, creating problems of overlap and cancelation across participants). However, we recognize that the Tb and P60 responses are only a relatively small part of the neurophysiological responses to these stimuli.

Another limitation of the present study is that auditory ERPs were recorded in response to broad band stimuli, whereas psychophysical thresholds were measured in response to pure tones. This may complicate interpretation of the correlation between Tb amplitudes and hearing thresholds.

Indeed, the ERP task in the present study was originally intended to investigate multisensory integration (by comparing multisensory and summed unisensory responses, as in, e.g., Giard and Peronnet, 1999; Foxe et al., 2000; Russo et al., 2010; Brandwein et al., 2011). For this reason, we did not specifically design it to explore neural or attentional processes relevant to autistic sensory experiences in a naturalistically valid manner.

The present study sample is also drawn from a relatively limited range of the autistic population. Due to a recruitment restriction which made collection of self-report and laboratory task measures easier, participants almost universally did not have intellectual disabilities. Few female and no non-binary participants were recruited. Due to another recruitment restriction intended to minimize variance associated with developmental change, a limited age range (Tomchek and Dunn, 2007; Cascio et al., 2008; Tavassoli et al., 2014; Fukuyama et al., 2017) was used. While the narrowness of this sample has some advantages, it reduces the generalizability of these results.

Additionally, due to the small size of the TD sample, we did not have sufficient power to explore correlation effects in that group.

Finally, the present study’s psychophysical tasks did not present catch/null trials with no stimuli. Thus, we cannot rule out the possibility that perceptual conservatism may have inflated our estimates of some participants’ sensory thresholds (Quinde-Zlibut et al., 2020).

## Conclusion

The present study provides a rigorous multidimensional examination of sensory processing in ASD, using neurophysiological, psychophysical, self-report, and parent-report measures. The most robust group differences were observed on questionnaire measures, which may therefore have particular value in identifying sensory challenges and discomfort in community settings. The SEQ, a measure designed for autism, appeared to perform particularly well, emphasizing the advantages of using measures designed with autistic sensory phenotypes in mind. We observed no group differences in psychophysical thresholds, and a non-significant trend toward lower auditory thresholds in ASD from the left ear might reflect attentional or other processes rather than acuity *per se*. We observed no associations between sensory acuity and questionnaire measures. Future research should continue exploring ways of increasing the naturalistic validity of laboratory-based tasks, and future autism research should carefully consider the types of laboratory-based sensory measures most likely to tap into atypicalities in autistic sensory experiences.

We also observed some group differences in ERP measures. Although findings of attenuated somatosensory P60 amplitudes and delayed auditory Tb latencies in the ASD group were no longer significant after correlating for cognitive ability, they remained as trends, and might reflect genuine group differences.

Furthermore, in the ASD group, we observed a correlation between auditory Tb amplitudes and auditory thresholds, such that participants with higher auditory thresholds/reduced acuity had larger Tb responses to auditory stimuli. This effect might reflect compensatory processing and/or prolonged maintenance of sensory representations, but replication and further research will be important to fully understand this effect. Nevertheless, the mere existence of the effect implies that hearing thresholds could be an important confound for studies exploring functional brain responses to auditory stimulation. Measurement of hearing thresholds in such studies might help researchers further investigate and control for any confounding effects.

## Data Availability Statement

The raw data supporting the conclusions of this article will be made available by the authors on request, without undue reservation.

## Ethics Statement

The studies involving human participants were reviewed and approved by UC Davis Institutional Review Board. Written informed consent to participate in this study was provided by the participants’ legal guardian/next of kin.

## Author Contributions

SR, CS, and YT designed the present study. YT, IZ, SR, and CS contributed to data collection and processing. PD analyzed the data and drafted this manuscript, which was read, edited, and approved by all authors. All authors contributed to the article and approved the submitted version.

## Conflict of Interest

The authors declare that the research was conducted in the absence of any commercial or financial relationships that could be construed as a potential conflict of interest.

## Publisher’s Note

All claims expressed in this article are solely those of the authors and do not necessarily represent those of their affiliated organizations, or those of the publisher, the editors and the reviewers. Any product that may be evaluated in this article, or claim that may be made by its manufacturer, is not guaranteed or endorsed by the publisher.

## Footnotes

1 There is an ongoing debate in the autism world regarding whether person-first (i.e., “person with autism”) or identity-first (i.e., “autistic person”) language should be used to describe autism. In this paper, we have chosen to use identity-first language. Although not all individuals on the autism spectrum prefer identity-first language, many do (Kenny et al., 2016; Bury et al., 2020). Furthermore, some arguments suggest that person-first language may reflect and/or accentuate stigma towards autism (Sinclair, 2013; Gernsbacher, 2017). We have also chosen to use the term “Autism Spectrum Development” as an alternative to “Autism Spectrum Disorder” or “Autism Spectrum Condition,” terms which are not widely endorsed by autistic individuals (Kenny et al., 2016). The phrase “development” is also a neutral descriptor, whereas the term “disorder,” and perhaps the term “condition” as well, appear to suggest subjective value judgments that are arguably unnecessary and unhelpful in scientific research.

2
www.easycap.de

## References

[B1] Albrecht R. Suchodoletz W. v. Uwer R. (2000). The development of auditory evoked dipole source activity from childhood to adulthood. *Clin. Neurophysiol.* 111 2268–2276. 10.1016/s1388-2457(00)00464-8 11090781

[B2] Ausderau K. Sideris J. Furlong M. Little L. M. Bulluck J. Baranek G. T. (2014). National survey of sensory features in children with ASD: factor structure of the sensory experience questionnaire (3.0). *J. Autism Dev. Disord.* 44 915–925. 10.1007/s10803-013-1945-1 24097141 PMC3949144

[B3] Baranek G. T. (1999). Autism during infancy: a retrospective video analysis of sensory-motor and social behaviors at 9-12 months of age. *J. Autism Dev. Disord.* 29 213–224. 10.1023/a:1023080005650 10425584

[B4] Baranek G. T. David F. J. Poe M. D. Stone W. L. Watson L. R. (2006). Sensory Experiences Questionnaire: discriminating sensory features in young children with autism, developmental delays, and typical development. *J. Child Psychol. Psychiatry Allied Discip.* 47 591–601. 10.1111/j.1469-7610.2005.01546.x 16712636

[B5] Baranek G. T. Woynaroski T. G. Nowell S. Turner-Brown L. DuBay M. Crais E. R. (2018). Cascading effects of attention disengagement and sensory seeking on social symptoms in a community sample of infants at-risk for a future diagnosis of autism spectrum disorder. *Dev. Cogn. Neurosci.* 29 30–40. 10.1016/j.dcn.2017.08.006 28869201 PMC6414208

[B6] Berument S. K. Rutter M. Lord C. Pickles A. Bailey A. (1999). Autism screening questionnaire: diagnostic validity. *Br. J. Psychiatry* 175 444–451. 10.1192/bjp.175.5.444 10789276

[B7] Bießmann F. Rukat T. Schmidt P. Naidu P. Schelter S. Taptunov A. (2019). DataWig: missing value imputation for tables. *J. Mach. Learn. Res.* 20 1–6.

[B8] Blakemore S. Tavassoli T. Calò S. Thomas R. M. Catmur C. Frith U. (2006). Tactile sensitivity in Asperger syndrome. *Brain Cogn.* 61 5–13. 10.1016/j.bandc.2005.12.013 16500009

[B9] Bleyenheuft Y. Cols C. Arnould C. Thonnard J. (2006). Age-related changes in tactile spatial resolution from 6 to 16 years old. *Somatosens. Mot. Res.* 23 83–87. 10.1080/08990220600816440 17178543

[B10] Bleyenheuft Y. Wilmotte P. Thonnard J.-L. (2010). Relationship between tactile spatial resolution and digital dexterity during childhood. *Somatosens. Mot. Res.* 27 9–14. 10.3109/08990220903471831 20141405

[B11] Blomberg R. Capusan A. J. Signoret C. Danielsson H. Rönnberg J. (2021). The effects of working memory load on auditory distraction in adults with attention deficit hyperactivity disorder. *Front. Hum. Neurosci.* 15:771711. 10.3389/fnhum.2021.771711 34916918 PMC8670091

[B12] Bonnel A. McAdams S. Smith B. Berthiaume C. Bertone A. Ciocca V. (2010). Enhanced pure-tone pitch discrimination among persons with autism but not Asperger syndrome. *Neuropsychologia* 48 2465–2475. 10.1016/j.neuropsychologia.2010.04.020 20433857

[B13] Brandwein A. B. Foxe J. J. Russo N. N. Altschuler T. S. Gomes H. Molholm S. (2011). The development of audiovisual multisensory integration across childhood and early adolescence: a high-density electrical mapping study. *Cereb. Cortex* 21 1042–1055. 10.1093/cercor/bhq170 20847153 PMC3077428

[B14] Brown C. Dunn W. (2002). *Adult/Adolescent Sensory Profile: User’s manual.* San Antonio, TX: Psychological Corporation.

[B15] Brunet D. Murray M. M. Michel C. M. (2011). Spatiotemporal analysis of multichannel EEG: CARTOOL. *Comput. Intell. Neurosci.* 2011:813870. 10.1155/2011/813870 21253358 PMC3022183

[B16] Bury S. M. Jellett R. Spoor J. R. Hedley D. (2020). “It defines who I am” or “it’s something I have”: what language do [autistic] Australian adults [on the autism spectrum] prefer? *J. Autism Dev. Disord.* 10.1007/s10803-020-04425-3 [Epub ahead of print]. 32112234

[B17] Campbell J. Sharma A. (2013). Compensatory changes in cortical resource allocation in adults with hearing loss. *Front. Syst. Neurosci.* 7:71. 10.3389/fnsys.2013.00071 24478637 PMC3905471

[B18] Cascio C. J. Gu C. Schauder K. B. Key A. P. Yoder P. (2015). Somatosensory event-related potentials and association with tactile behavioral responsiveness patterns in children with ASD. *Brain Topogr.* 28 895–903. 10.1007/s10548-015-0439-1 26016951 PMC4601930

[B19] Cascio C. J. McGlone F. Folger S. Tannan V. Baranek G. Pelphrey K. A. (2008). Tactile perception in adults with autism: a multidimensional psychophysical study. *J. Autism Dev. Disord.* 38 127–137. 10.1007/s10803-007-0370-8 17415630 PMC2185746

[B20] Chien Y. L. Hsieh M. H. Gau S. S. F. (2017). Mismatch negativity and P3a in adolescents and young adults with autism spectrum disorders: behavioral correlates and clinical implications. *J. Autism Dev. Disord.* 48 1684–1697. 10.1007/s10803-017-3426-4 29198040

[B21] Cliff N. (1993). Dominance statistics: ordinal analyses to answer ordinal questions. *Quant. Methods Psychol.* 114 494–509. 10.1037/0033-2909.114.3.494

[B22] Cox R. M. Moore J. N. (1988). Composite speech spectrum for hearing and gain prescriptions. *J. Speech Lang. Hear. Res.* 31 102–107. 10.1044/jshr.3101.102 3352247

[B23] Crane L. Goddard L. Pring L. (2009). Sensory processing in adults with autism spectrum disorders. *Autism* 13 215–228. 10.1177/1362361309103794 19369385

[B24] Damiano-Goodwin C. R. Woynaroski T. G. Simon D. M. Ibañez L. V. Murias M. Kirby A. (2018). Developmental sequelae and neurophysiologic substrates of sensory seeking in infant siblings of children with autism spectrum disorder. *Dev. Cogn. Neurosci.* 29 41–53. 10.1016/j.dcn.2017.08.005 28889988 PMC5812859

[B25] Demopoulos C. Lewine J. D. (2016). Audiometric profiles in autism spectrum disorders: does subclinical hearing loss impact communication? *Autism Res.* 9 107–120. 10.1002/aur.1495 25962745 PMC4641833

[B26] Demopoulos C. Yu N. Tripp J. Mota N. Brandes-Aitken A. N. Desai S. S. (2017). Magnetoencephalographic imaging of auditory and somatosensory cortical responses in children with autism and sensory processing dysfunction. *Front. Hum. Neurosci.* 11:259. 10.3389/fnhum.2017.00259 28603492 PMC5445128

[B27] Donkers F. C. L. Carlson M. Schipul S. E. Belger A. Baranek G. T. (2020). Auditory event-related potentials and associations with sensory patterns in children with autism spectrum disorder, developmental delay, and typical development. *Autism* 24 1093–1110. 10.1177/1362361319893196 31845589 PMC7297652

[B28] Donkers F. C. L. Schipul S. E. Baranek G. T. Cleary K. M. Willoughby M. T. Evans A. M. (2015). Attenuated auditory event-related potentials and associations with atypical sensory response patterns in children with autism. *J. Autism Dev. Disord.* 45 506–523. 10.1007/s10803-013-1948-y 24072639 PMC4556131

[B29] Dunn W. (1997). The impact of sensory processing abilities on the daily lives of young children and their families: a conceptual model. *Infants Young Child.* 9 23–35. 10.1097/00001163-199704000-00005

[B30] Dunn W. (1999). *The Sensory Profile: User’s Manual.* San Antonio, TX: Psychological Corporation.

[B31] Dwyer P. Wang X. De Meo-Monteil R. Hsieh F. Saron C. D. Rivera S. M. (2020). Defining clusters of young autistic and typically developing children based on loudness-dependent auditory electrophysiological responses. *Mol. Autism* 11:48. 10.1186/s13229-020-00352-3 32539866 PMC7294610

[B32] Ehlers S. Gillberg C. Wing L. (1999). A screening questionnaire for Asperger syndrome and other high- functioning autism spectrum disorders in school age children. *J. Autism Dev. Disord.* 29 129–141. 10.1023/a:1023040610384 10382133

[B33] Eimer M. Maravita A. Van Velzen J. Husain M. Driver J. (2002). The electrophysiology of tactile extinction: ERP correlates of unconscious somatosensory processing. *Neuropsychologia* 40 2438–2447. 10.1016/s0028-3932(02)00079-9 12417471

[B34] Elwin M. Ek L. Kjellin L. Schröder A. (2013). Too much or too little: hyper- and hypo-reactivity in high-functioning autism spectrum conditions. *J. Intellect. Dev. Disabil.* 38 232–241. 10.3109/13668250.2013.815694 23984882

[B35] Enzler F. Fournier P. Noreña A. J. (2021a). A psychoacoustic test for diagnosing hyperacusis based on ratings of natural sounds. *Hear. Res.* 400:108124. 10.1016/j.heares.2020.108124 33321385

[B36] Enzler F. Loriot C. Fournier P. Noreña A. J. (2021b). A psychoacoustic test for misophonia assessment. *Sci. Rep.* 11:11044. 10.1038/s41598-021-90355-8 34040061 PMC8155015

[B37] Fjell A. M. Walhovd K. B. (2003). Effects of auditory stimulus intensity and hearing threshold on the relationship among P300, age, and cognitive function. *Clin. Neurophysiol.* 114 799–807. 10.1016/s1388-2457(03)00030-0 12738426

[B38] Foxe J. J. Morocz I. A. Murray M. M. Higgins B. A. Javitt D. C. Schroeder C. E. (2000). Multisensory auditory-somatosensory interactions in early cortical processing revealed by high-density electrical mapping. *Cogn. Brain Res.* 10 77–83. 10.1016/s0926-6410(00)00024-0 10978694

[B39] Fukuyama H. Kumagaya S. I. Asada K. Ayaya S. Kato M. (2017). Autonomic versus perceptual accounts for tactile hypersensitivity in autism spectrum disorder. *Sci. Rep.* 7:8259. 10.1038/s41598-017-08730-3 PMC5557757 28811601

[B40] Gaetz W. Jurkiewicz M. T. Kilaru S. Blaskey L. Schwartz E. S. Roberts T. P. L. (2017). Neuromagnetic responses to tactile stimulation of the fingers: evidence for reduced cortical inhibition for children with Autism Spectrum Disorder and children with epilepsy. *NeuroImage Clin.* 16 624–633. 10.1016/j.nicl.2017.06.026 28971012 PMC5619996

[B41] Gernsbacher M. A. (2017). Editorial perspective: the use of person-first language in scholarly writing may accentuate stigma. *J. Child Psychol. Psychiatry* 58 859–861. 10.1111/jcpp.12706 28621486 PMC5545113

[B42] Ghent L. (1961). Developmental changes in tactual thresholds on dominant and nondominant sides. *J. Comp. Physiol. Psychol.* 54 670–673. 10.1037/h0047319 13898184

[B43] Giard M. H. Peronnet F. (1999). Auditory-visual integration during multimodal object recognition in humans: a behavioral and electrophysiological study. *J. Cogn. Neurosci.* 11 473–490. 10.1162/089892999563544 10511637

[B44] Gilley P. M. Sharma A. Dorman M. Martin K. (2005). Developmental changes in refractoriness of the cortical auditory evoked potential. *Clin. Neurophysiol.* 116 648–657. 10.1016/j.clinph.2004.09.009 15721079

[B45] Gondan M. Minakata K. (2016). A tutorial on testing the race model inequality. *Atten. Percept. Psychophys.* 78 723–735. 10.3758/s13414-015-1018-y 26637234

[B46] Goossens T. Vercammen C. Wouters J. van Wieringen A. (2019). The association between hearing impairment and neural envelope encoding at different ages. *Neurobiol. Aging* 74 202–212. 10.1016/j.neurobiolaging.2018.10.008 30472387

[B47] Gotham K. Pickles A. Lord C. (2009). Standardizing ADOS scores for a measure of severity in autism spectrum disorders. *J. Autism Dev. Disord.* 39, 693–705. 19082876 10.1007/s10803-008-0674-3 PMC2922918

[B48] Gotham K. Risi S. Pickles A. Lord C. (2007). The Autism Diagnostic Observation Schedule: revised algorithms for improved diagnostic validity. *J. Autism Dev. Disord.* 37 613–627. 10.1007/s10803-006-0280-1 17180459

[B49] Gravel J. S. Dunn M. Lee W. W. Ellis M. A. (2006). Peripheral audition of children on the autistic spectrum. *Ear Hear.* 27 299–312. 10.1097/01.aud.0000215979.65645.22 16672798

[B50] Haesen B. Boets B. Wagemans J. (2011). A review of behavioural and electrophysiological studies on auditory processing and speech perception in autism spectrum disorders. *Res. Autism Spectr. Disord.* 5 701–714. 10.1016/s0387-7604(02)00191-2 12689694

[B51] Haigh S. M. (2018). Variable sensory perception in autism. *Eur. J. Neurosci.* 47 602–609. 10.1111/ejn.13601 28474794

[B52] Hansen H. A. Leber A. B. Saygin Z. M. (2021). What sound sources trigger misophonia? Not just chewing and breathing. *J. Clin. Psychol.* 77 2609–2625. 10.1002/jclp.23196 34115383

[B53] Hoth S. (1993). Computer-aided hearing threshold determination from cortical auditory evoked potentials. *Scand. Audiol.* 22 165–177. 10.3109/01050399309047463 8210956

[B54] Hudac C. M. DesChamps T. D. Arnett A. B. Cairney B. E. Ma R. Webb S. J. (2018). Early enhanced processing and delayed habituation to deviance sounds in autism spectrum disorder. *Brain Cogn.* 123 110–119. 10.1016/j.bandc.2018.03.004 29550506 PMC5893357

[B55] Hus V. Lord C. (2014). The autism diagnostic observation schedule, module 4: revised algorithm and standardized severity scores. *J. Autism Dev. Disord.* 44 1996–2012. 10.1007/s10803-014-2080-3 24590409 PMC4104252

[B56] Ide M. Yaguchi A. Sano M. Fukatsu R. Wada M. (2019). Higher tactile temporal resolution as a basis of hypersensitivity in individuals with autism spectrum disorder. *J. Autism Dev. Disord.* 49 44–53. 10.1007/s10803-018-3677-8 30019275 PMC6331495

[B57] Jones C. R. G. Happé F. Baird G. Simonoff E. Marsden A. J. S. Tregay J. (2009). Auditory discrimination and auditory sensory behaviours in autism spectrum disorders. *Neuropsychologia* 47 2850–2858. 10.1016/j.neuropsychologia.2009.06.015 19545576

[B58] Karhson D. S. Golob E. J. (2016). Atypical sensory reactivity influences auditory attentional control in adults with autism spectrum disorders. *Autism Res.* 9 1079–1092. 10.1002/aur.1593 26778164

[B59] Keehn B. Nair A. Lincoln A. J. Townsend J. Müller R. A. (2016). Under-reactive but easily distracted: an fMRI investigation of attentional capture in autism spectrum disorder. *Dev. Cogn. Neurosci.* 17 46–56. 10.1016/j.dcn.2015.12.002 26708773 PMC4728050

[B60] Keith J. M. Jamieson J. P. Bennetto L. (2019). The importance of adolescent self-report in autism spectrum disorder: integration of questionnaire and autonomic measures. *J. Abnorm. Child Psychol.* 47 741–754. 10.1007/s10802-018-0455-1 30073571 PMC6359986

[B61] Kenny L. Hattersley C. Molins B. Buckley C. Povey C. Pellicano E. (2016). Which terms should be used to describe autism? Perspectives from the UK autism community. *Autism* 20 442–462. 10.1177/1362361315588200 26134030

[B62] Kern J. K. Trivedi M. H. Garver C. R. Grannemann B. D. Andrews A. A. Savla J. S. (2006). The pattern of sensory processing abnormalities in autism. *Autism* 10 480–494. 10.1177/1362361306066564 16940314

[B63] Khalfa S. Bruneau N. Rogé B. Georgieff N. Veuillet E. Adrien J.-L. (2004). Increased perception of loudness in autism. *Hear. Res.* 198 87–92. 10.1016/j.heares.2004.07.006 15617227

[B64] Kientz M. A. Dunn W. (1997). A comparison of the performance of children with and without autism on the Sensory Profile. *Am. J. Occup. Ther.* 51 530–537. 10.5014/ajot.51.7.530 9242859

[B65] Kolesnik A. Ali J. B. Gliga T. Guiraud J. Charman T. Jones E. J. H. (2019). Increased cortical reactivity to repeated tones at 8 months in infants with later ASD. *Transl. Psychiatry* 9:46. 10.1038/s41398-019-0393-x 30700699 PMC6353960

[B66] Kolodny T. Schallmo M.-P. Gerdts J. Edden R. A. E. Bernier R. A. Murray S. O. (2020). Concentrations of cortical GABA and glutamate in young adults with autism spectrum disorder. *Autism Res.* 13 1111–1129. 10.1002/aur.2300 32297709 PMC7387217

[B67] Kuiper M. W. M. Verhoeven E. W. M. Geurts H. M. (2019). Stop making noise! Auditory sensitivity in adults with an autism spectrum disorder diagnosis: physiological habituation and subjective detection thresholds. *J. Autism Dev. Disord.* 49 2116–2128. 10.1007/s10803-019-03890-9 30680585 PMC6483953

[B68] Lightfoot G. (2016). Summary of the N1-P2 cortical auditory evoked potential to estimate the auditory threshold in adults. *Semin. Hear.* 37 1–8. 10.1055/s-0035-1570334 27587918 PMC4910570

[B69] Lin L.-Y. Huang P.-C. (2019). Quality of life and its related factors for adults with autism spectrum disorder. *Disabil. Rehabil.* 41 896–903. 10.1080/09638288.2017.1414887 29228834

[B70] Lopez-Calderon J. Luck S. J. (2014). ERPLAB: an open-source toolbox for the analysis of event-related potentials. *Front. Hum. Neurosci.* 8:213. 10.3389/fnhum.2014.00213 24782741 PMC3995046

[B71] Lord C. Risi S. Linda L. Cook E. H. Jr. Leventhal B. L. DiLavore P. C. (2000). The Autism Diagnostic Observation Schedule - Generic: a standard measure of social and communication deficits associated with the spectrum of autism. *J. Autism Dev. Disord.* 30 205–223. 11055457

[B72] Lord C. Rutter M. Le Couteur A. (1994). Autism diagnostic interview-revised: a revised version of a diagnostic interview for caregivers of individuals with possible pervasive developmental disorders. *J. Autism Dev. Disord.* 24 659–685. 10.1007/BF02172145 7814313

[B73] Luck S. J. (2014). *An Introduction to the Event-Related Potential Technique*, 2nd Edn. Cambridge, MA: MIT Press.

[B74] Ludlow A. Mohr B. Whitmore A. Garagnani M. Pulvermüller F. Gutierrez R. (2014). Auditory processing and sensory behaviours in children with autism spectrum disorders as revealed by mismatch negativity. *Brain Cogn.* 86 55–63. 10.1016/j.bandc.2014.01.016 24565813

[B75] Maeder P. P. Meuli R. A. Adriani M. Bellmann A. Fornari E. Thiran J.-P. (2001). Distinct pathways involved in sound recognition and localization: a human fMRI study. *Neuroimage* 14 802–816. 10.1006/nimg.2001.0888 11554799

[B76] Marco E. J. Khatibi K. Hill S. S. Siegel B. Arroyo M. S. Dowling A. F. (2012). Children with autism show reduced somatosensory response: an MEG study. *Autism Res.* 5 340–351. 10.1002/aur.1247 22933354 PMC3474892

[B77] Mayer J. L. (2017). The relationship between autistic traits and atypical sensory functioning in neurotypical and ASD adults: a spectrum approach. *J. Autism Dev. Disord.* 47 316–327. 10.1007/s10803-016-2948-5 27848052

[B78] Mayer J. L. Hannent I. Heaton P. F. (2016). Mapping the developmental trajectory and correlates of enhanced pitch perception on speech processing in adults with ASD. *J. Autism Dev. Disord.* 46 1562–1573. 10.1007/s10803-014-2207-6 25106823

[B79] McConachie H. Wilson C. Mason D. Garland D. Parr J. R. Rattazzi A. (2020). What is important in measuring quality of life? Reflections by autistic adults in four countries. *Autism Adulthood* 2 4–12. 10.1089/aut.2019.0008 PMC8992842 36600984

[B80] Näätänen R. Kreegipuu K. (2011). “The mismatch negativity (MNN),” in *The Oxford Handbook of Event-Related Potential Components*, eds Kappenman E. S. Luck S. J. (Oxford: Oxford University Press), 143–157. 10.3389/fnhum.2014.00729

[B81] Nader A.-M. Courchesne V. Dawson M. Soulières I. (2016). Does WISC-IV underestimate the intelligence of autistic children? *J. Autism Dev. Disord.* 46 1582–1589. 10.1007/s10803-014-2270-z 25308198

[B82] Nader A.-M. Jelenic P. Soulières I. (2015). Discrepancy between WISC-III and WISC-IV cognitive profile in autism spectrum: What does it reveal about autistic cognition? *PLoS One* 10:e0144645. 10.1371/journal.pone.0144645 26673881 PMC4686055

[B83] O’Connor K. (2012). Auditory processing in autism spectrum disorder: A review. *Neurosci. Biobehav. Rev.* 36 836–854. 10.1016/j.neubiorev.2011.11.008 22155284

[B84] Occelli V. Esposito G. Venuti P. Arduino G. M. Zampini M. (2013). Attentional shifts between audition and vision in autism spectrum disorders. *Res. Autism Spectr. Disord.* 7 517–525. 10.1016/j.rasd.2012.12.003

[B85] Oldfield R. C. (1971). The assessment and analysis of handedness: the Edinburgh inventory. *Neuropsychologia* 9 97–113. 10.1016/0028-3932(71)90067-4 5146491

[B86] Perrin F. Pernier J. Bertrand O. Giard M. Echallier J. (1987). Mapping of scalp potentials by surface spline interpolation. *Electroencephalogr. Clin. Neurophysiol.* 66 75–81. 10.1016/0013-4694(87)90141-6 2431869

[B87] Peters R. M. Goldreich D. (2013). Tactile spatial acuity in childhood: effects of age and fingertip size. *PLoS One* 8:e84650. 10.1371/journal.pone.0084650 24454612 PMC3891499

[B88] Pfefferbaum A. (1990). Model estimates of CSF and skull influences on scalp-recorded ERPs. *Alcohol* 7 479–482. 10.1016/0741-8329(90)90035-b 2222852

[B89] Pollock V. E. Schneider L. S. (1992). P3 from auditory stimuli in healthy elderly subjects: hearing threshold and tone stimulus frequency. *Int. J. Psychophysiol.* 12 237–241. 10.1016/0167-8760(92)90062-g 1639670

[B90] Ponton C. Eggermont J. Khosla D. Kwong B. Don M. (2002). Maturation of human central auditory system activity: separating auditory evoked potentials by dipole source modeling. *Clin. Neurophysiol.* 113 407–420. 10.1016/s1388-2457(01)00733-7 11897541

[B91] Pratt H. (2011). “04. Sensory ERP components,” in *The Oxford Handbook of Event-Related Potential Components*, eds Kappenman E. S. Luck S. J. (New York, NY: Oxford University Press).

[B92] Puts N. A. J. Wodka E. L. Tommerdahl M. Mostofsky S. H. Edden R. A. E. (2014). Impaired tactile processing in children with autism spectrum disorder. *J. Neurophysiol.* 111 1803–1811. 10.1152/jn.00890.2013 24523518 PMC4044368

[B93] Quinde-Zlibut J. M. Okitondo C. D. Williams Z. J. Weitlauf A. Mash L. E. Heflin B. H. (2020). Elevated thresholds for light touch in children with autism reflect more conservative perceptual decision-making rather than a sensory deficit. *Front. Hum. Neurosci.* 14:122. 10.3389/fnhum.2020.00122 32317953 PMC7154145

[B94] Rauschecker J. P. Tian B. (2004). Processing of band-passed noise in the lateral auditory belt cortex of the rhesus monkey. *J. Neurophysiol.* 91 2578–2589. 10.1152/jn.00834.2003 15136602

[B95] Remington A. Fairnie J. (2017). A sound advantage: increased auditory capacity in autism. *Cognition* 166 459–465. 10.1016/j.cognition.2017.04.002 28476357

[B96] Roberts T. P. L. Bloy L. Ku M. Blaskey L. Jackel C. R. Edgar J. C. (2020). A multimodal study of the contributions of conduction velocity to the auditory evoked neuromagnetic response: anomalies in autism spectrum disorder. *Autism Res.* 13 1730–1745. 10.1002/aur.2369 32924333

[B97] Roberts T. P. L. Lanza M. R. Della J. Qasmieha S. Hines K. Blaskey L. (2013). Maturational differences in thalamocortical white matter microstructure and auditory evoked response latencies in autism spectrum disorders. *Brain Res.* 1537 79–85. 10.1016/j.brainres.2013.09.011 24055954 PMC3970268

[B98] Russo N. Foxe J. J. Brandwein A. B. Altschuler T. Gomes H. Molholm S. (2010). Multisensory processing in children with autism: high-density electrical mapping of auditory-somatosensory integration. *Autism Res.* 3 253–267. 10.1002/aur.152 20730775

[B99] Saggar M. King B. G. Zanesco A. P. MacLean K. A. Aichele S. R. Jacobs T. L. (2012). Intensive training induces longitudinal changes in meditation state-related EEG oscillatory activity. *Front. Hum. Neurosci.* 6:256. 10.3389/fnhum.2012.00256 22973218 PMC3437523

[B100] Sapey-Triomphe L.-A. Lamberton F. Sonié S. Mattout J. Schmitz C. (2019). Tactile hypersensitivity and GABA concentration in the sensorimotor cortex of adults with autism. *Autism Res.* 12 562–575. 10.1002/aur.2073 30632707

[B101] Schubert R. Blankenburg F. Lemm S. Villringer A. Curio G. (2006). Now you feel it - now you don’t: ERP correlates of somatosensory awareness. *Psychophysiology* 43 31–40. 10.1111/j.1469-8986.2006.00379.x 16629683

[B102] Schulz S. E. Stevenson R. A. (2021). Convergent validity of behavioural and subjective sensitivity in relation to autistic traits. *J. Autism Dev. Disord.* 52 758–770. 10.1007/s10803-021-04974-1 33770325

[B103] Schwartz S. Shinn-Cunningham B. Tager-Flusberg H. (2018). Meta-analysis and systematic review of the literature characterizing auditory mismatch negativity in individuals with autism. *Neurosci. Biobehav. Rev.* 87 106–117. 10.1016/j.neubiorev.2018.01.008 29408312 PMC5845770

[B104] Schwartz S. Wang L. Shinn-Cunningham B. G. Tager-Flusberg H. (2020a). Atypical perception of sounds in minimally and low verbal children and adolescents with autism as revealed by behavioral and neural measures. *Autism Res.* 13 1718–1729. 10.1002/aur.2363 32881387

[B105] Schwartz S. Wang L. Shinn-Cunningham B. G. Tager-Flusberg H. (2020b). Neural evidence for speech processing deficits during a cocktail party scenario in minimally and low verbal adolescents and young adults with autism. *Autism Res.* 13 1828–1842. 10.1002/aur.2356 32827357

[B106] Sharma A. Kraus N. McGee T. J. Nicol T. G. (1997). Developmental changes in P1 and N1 central auditory responses elicited by consonant-vowel syllables. *Electroencephalogr. Clin. Neurophysiol.* 104 540–545. 10.1016/s0168-5597(97)00050-6 9402896

[B107] Sinclair J. (2013). Why I dislike “Person First” language. *Auton. Crit. J. Interdiscip. Autism Stud.* 1 2–3. 10.1002/ab.21824 30706945 PMC6590429

[B108] Stoelting Co (2001). *Touch Test™ Sensory Evaluators: Semmes Weinstein Von Frey Aesthesiometers.* Wood Dale, IL: Stoelting Co

[B109] Stoelting Co (2021). *JVP Domes [Internet]*. Available online at: https://www.stoeltingco.com/jvp-domes.html (accessed September 30, 2021).

[B110] Takayama Y. Hashimoto R. Tani M. Kanai C. Yamada T. Watanabe H. (2014). Standardization of the Japanese version of the Glasgow Sensory Questionnaire (GSQ). *Res. Autism Spectr. Disord.* 8 347–353. 10.1016/j.rasd.2013.12.017

[B111] Tavassoli T. Hoekstra R. A. Baron-Cohen S. (2014). The Sensory Perception Quotient (SPQ): development and validation of a new sensory questionnaire for adults with and without autism. *Mol. Autism* 5:29. 10.1186/2040-2392-5-29 24791196 PMC4005907

[B112] Tomchek S. D. Dunn W. (2007). Sensory processing in children with and without autism: a comparative study using the Short Sensory Profile. *Am. J. Occup. Ther.* 61 190–200. 10.5014/ajot.61.2.190 17436841

[B113] Uljarević M. Baranek G. Vivanti G. Hedley D. Hudry K. Lane A. (2017). Heterogeneity of sensory features in autism spectrum disorder: challenges and perspectives for future research. *Autism Res.* 10 703–710. 10.1002/aur.1747 28266796

[B114] United States Census Bureau (2019). *Population Estimates, July 1, 2019: Sacramento County, California; Sacramento city, California; California; Davis city, California.* Available online at: https://www.census.gov/quickfacts/fact/table/sacramentocountycalifornia,sacramentocitycalifornia,CA,daviscitycalifornia/PST045219 (accessed September 30, 2021).

[B115] Uppal N. Foxe J. J. Butler J. S. Acluche F. Molholm S. (2016). The neural dynamics of somatosensory processing and adaptation across childhood: a high-density electrical mapping study. *J. Neurophysiol.* 115 1605–1619. 10.1152/jn.01059.2015 26763781 PMC4808123

[B116] Van Boven R. W. Johnson K. O. (1994). The limit of tactile spatial resolution in humans: grating orientation discrimination at the lip, tongue, and finger. *Neurology* 44 2361–2366. 10.1212/wnl.44.12.2361 7991127

[B117] Van Boven R. W. Hamilton R. H. Kauffman T. Keenan J. P. Pascual–Leone A. (2000). Tactile spatial resolution in blind Braille readers. *Neurology* 54 2230–2236. 10.1212/wnl.54.12.2230 10881245

[B118] Venker C. E. Mathée J. Neumann D. Edwards J. Saffran J. Weismer S. E. (2021). Competing perceptual salience in a visual word recognition task differentially affects children with and without autism spectrum disorder. *Autism Res.* 14 1147–1162. 10.1002/aur.2457 33372400 PMC8192461

[B119] Wechsler D. (2003). *The Wechsler Intelligence Scale for Children*, 4th Edn. San Antonio, TX: Psychological Corporation.

[B120] Weiland R. F. Polderman T. J. C. Hoekstra R. A. Smit D. J. A. Begeer S. (2020). The Dutch Sensory Perception Quotient-Short in adults with and without autism. *Autism* 24 2071–2080. 10.1177/1362361320942085 32720830 PMC7543020

[B121] Williams Z. J. Abdelmessih P. G. Key A. P. Woynaroski T. G. (2021a). Cortical auditory processing of simple stimuli is altered in autism: a meta-analysis of auditory evoked responses. *Biol. Psychiatry Cogn. Neurosci. Neuroimaging* 6 767–781. 10.1016/j.bpsc.2020.09.011 33229245 PMC8639293

[B122] Williams Z. J. Feldman J. I. Woynaroski T. G. (2021b). *Examining the Hierarchical Structure of Parent-Reported Sensory Features in Autism Using Bifactor Models. In: INSAR.* Available online at: https://www.autism-insar.org/resource/resmgr/docs/annualmeeting/Abstract_Book_INSAR2021Virtu.pdf (accessed January 1, 2022).

[B123] Wood E. T. Cummings K. K. Jung J. Patterson G. Okada N. Guo J. (2021). Sensory over-responsivity is related to GABAergic inhibition in thalamocortical circuits. *Transl. Psychiatry* 11:39. 10.1038/s41398-020-01154-0 33436538 PMC7804323

