# Supplementary material for: A Multidimensional Investigation of Sensory Processing in Autism: Parent- and Self-Report Questionnaires, Psychophysical Thresholds, and Event-Related Potentials in the Auditory and Somatosensory Modalities
Source: Front Hum Neurosci. 2022 May 10;16:811547. doi: 10.3389/fnhum.2022.811547 (PMC9127065; doi:10.3389/fnhum.2022.811547)
Supplement: Supplementary file 1 [file Data_Sheet_1.DOCX]

Supplementary Material

# Supplementary Figures


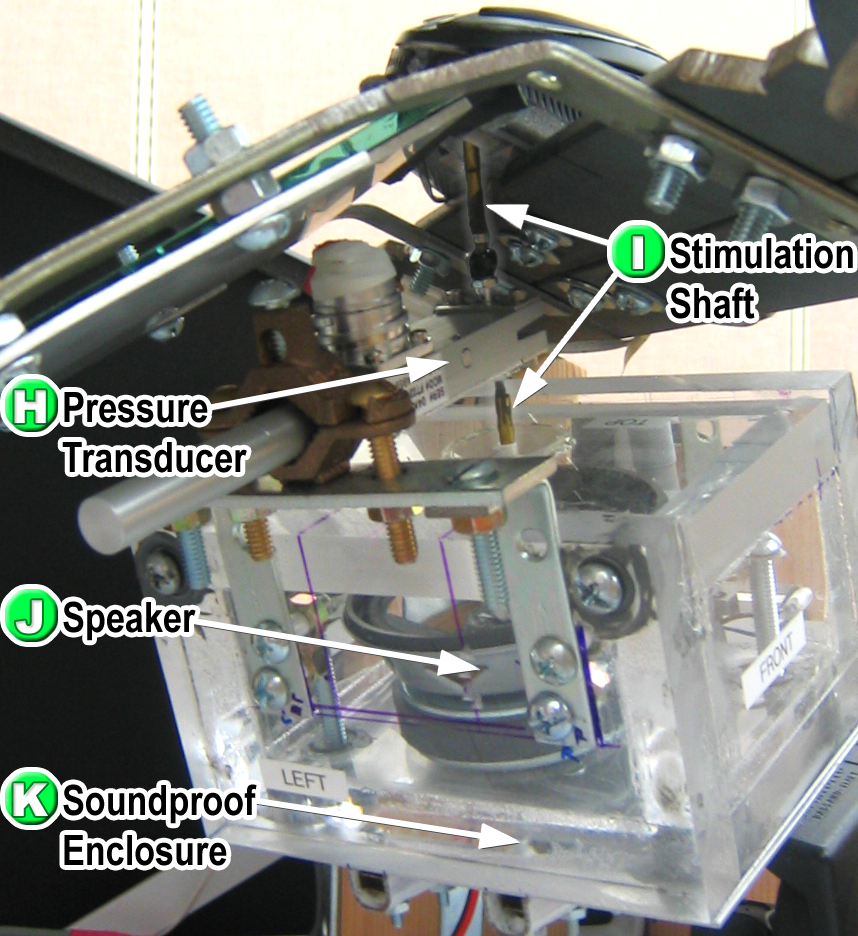


**Supplementary Figure 1.** Close-up of the somatosensory stimulator sub-assembly. The speaker (J) in a soundproof enclosure (K) drives a shaft (I) towards the stationary mouse. Finger pressure is recorded by a pressure transducer (H).


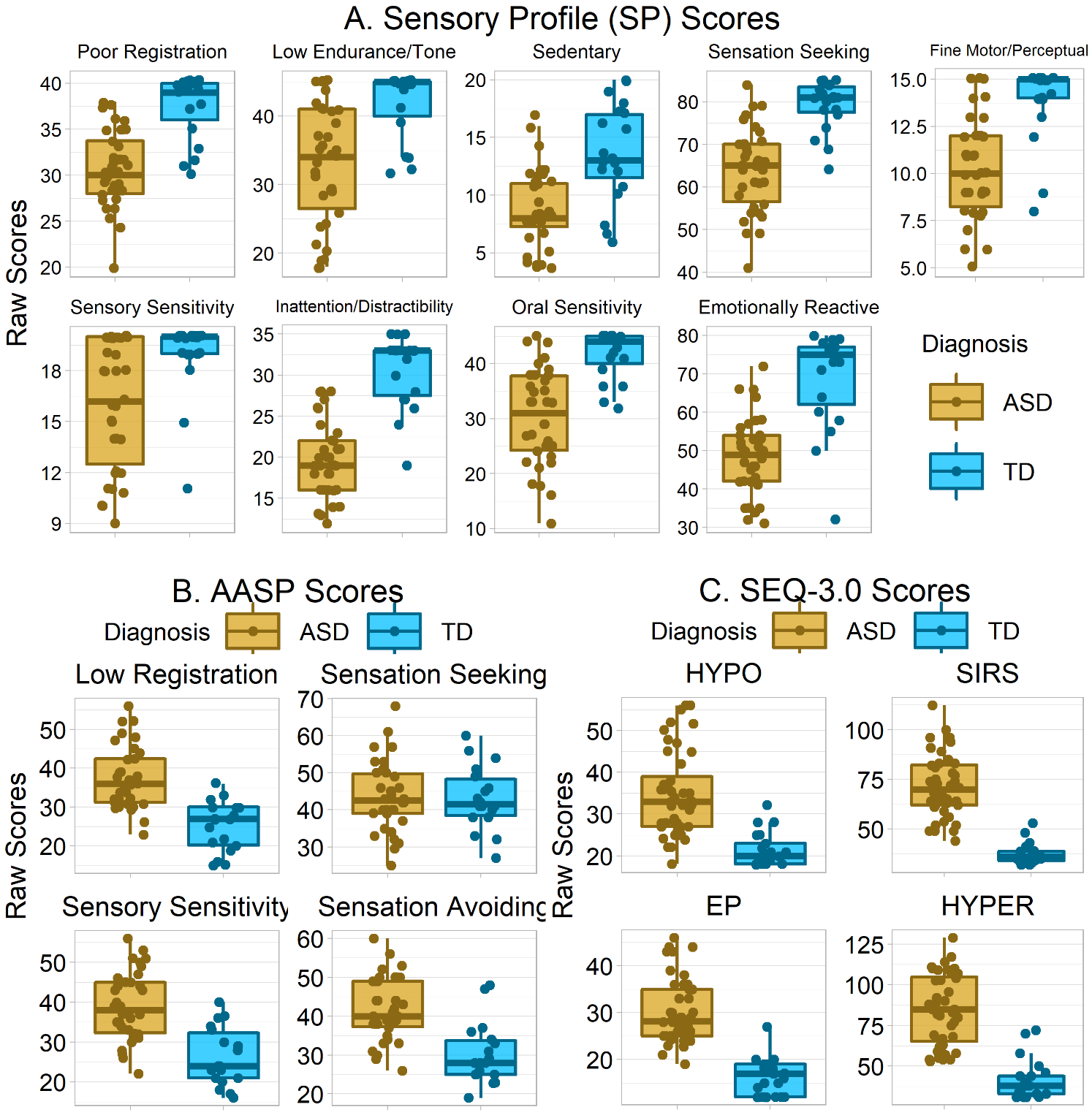


**Supplementary Figure 2.** Raw Sensory Profile (SP) scores (panel *A*, top), Adolescent-Adult Sensory Profile (AASP) scores (panel *B*, bottom left), and Sensory Experiences Questionnaire-3.0 (SEQ-3.0) scores (panel *C*, bottom right) in the autistic and typically-developing groups. Groups significantly differed in all patterns except AASP Sensation Seeking (see Supplementary Table 1). Lower scores on SP and higher scores on AASP and SEQ indicates greater levels of sensory patterns on each subscale.

# Supplementary Tables

| Supplementary Table 1. Results of statistical comparisons of autistic and typically-developing groups on SP, AASP, and SEQ scores. Scores represent the canonical factors of each measure as described by Dunn (1997), Brown and Dunn (2002), and Ausderau and colleagues (2014), respectively, summed across sensory modalities.  Results of ordinal Wilcoxon-Mann-Whitney tests are presented on the left, along with associated Cliff’s *δ* effect sizes. Lower scores on SP and higher scores on AASP and SEQ indicate greater levels of sensory patterns on each subscale. Thus, negative *δ* for SP and positive *δ* for AASP and SEQ indicate greater levels of sensory patterns in the ASD group than the TD group.  Results of ANCOVA analyses covarying for WISC PRI scores are presented on the right.  Corrected *p*-values employ the Holm-Bonferroni procedure to correct for seventeen comparisons (the total number of subscales across all three questionnaires), separately for ordinal and ANCOVA analyses. | | | | | | |
| --- | --- | --- | --- | --- | --- | --- |
|  | Wilcoxon-Mann-Whitney Effect | | | ANCOVA Effect | | |
|  | Cliff’s δ | *p* | Corrected *p* | *F* | *p* | Corrected *p* |
| SP Sensation Seeking | −.82 | < .0001 | < .0001 | 27.64 | < .0001 | < .0001 |
| SP Emotionally Reactive | −.78 | < .0001 | < .0001 | 30.35 | < .0001 | < .0001 |
| SP Low Endurance/Tone | −.63 | .0001 | .0006 | 10.62 | .002 | .006 |
| SP Oral Sensory Sensitivity | −.76 | < .0001 | < .0001 | 24.02 | < .0001 | < .0001 |
| SP Inattention/ Distractibility | −.88 | < .0001 | < .0001 | 61.63 | < .0001 | < .0001 |
| SP Poor Registration | −.80 | < .0001 | < .0001 | 27.69 | < .0001 | < .0001 |
| SP Sensory Sensitivity | −.46 | .004 | .009 | 7.48 | .009 | .02 |
| SP Sedentary | −.61 | .0002 | .0007 | 14.96 | .0003 | .002 |
| SP Fine Motor/Perceptual | −.70 | < .0001 | .0001 | 12.47 | .0009 | .004 |
|  |  |  |  |  |  |  |
| AASP Low Registration | .79 | < .0001 | < .0001 | 22.08 | < .0001 | .0002 |
| AASP Sensory Sensitivity | .73 | < .0001 | .0001 | 21.69 | < .0001 | .0002 |
| AASP Sensation Avoiding | .72 | < .0001 | .0001 | 14.45 | .0004 | .002 |
| AASP Sensation Seeking | .00 | .98 | .98 | 0.00 | .99 | .99 |
|  |  |  |  |  |  |  |
| SEQ HYPO | .83 | < .0001 | < .0001 | 27.25 | < .0001 | < .0001 |
| SEQ HYPER | .92 | < .0001 | < .0001 | 61.71 | < .0001 | < .0001 |
| SEQ SIRS | .98 | < .0001 | < .0001 | 86.31 | < .0001 | < .0001 |
| SEQ EP | .96 | < .0001 | < .0001 | 66.23 | < .0001 | < .0001 |

| Supplementary Table 2. Results of analyses conducted in the autistic group exploring correlations between psychophysical thresholds and sections of the AASP, SP, and SEQ in the same modality that examine low thresholds or enhanced perception. Note that the SEQ tactile enhanced perception subscale is excluded due to reliance on a single item. Pearson’s correlation coefficients and *p*-values are presented, along with Kendall’s ordinal equivalents; the latter are robust to outliers. *P*-values are given twice in each cell: above without correction and below with correction for seven comparisons per the Holm-Bonferroni procedure (separately for Pearson’s and Kendall’s correlations). | | | | |
| --- | --- | --- | --- | --- |
| Questionnaire Subscale | Pearson correlation | | Kendall’s correlation | |
|  | *r* | *p* | *τ* | *p* |
| Hearing Thresholds (Audiometry) | | | | |
| AASP Auditory Low Threshold | .06 | .76  >.99 | .02 | .88  .88 |
| SP Auditory Low Threshold | .02 | .92  .92 | .04 | .75  >.99 |
| SEQ Auditory EP | −.20 | .22  >.99 | −.10 | .38  >.99 |
| Von Frey Thresholds | | | | |
| AASP Tactile Low Threshold | −.28 | .20  >.99 | −.13 | .42  >.99 |
| SP Tactile Low Threshold | .11 | .57  >.99 | .11 | .41  >.99 |
| JVP Dome Thresholds | | | | |
| AASP Tactile Low Threshold Score | −.21 | .33  >.99 | −.19 | .23  >.99 |
| SP Tactile Low Threshold | .05 | .79  >.99 | .04 | .78  >.99 |

| Supplementary Table 3. Results of analyses conducted in the autistic group exploring correlations between auditory Tb ERP amplitudes and auditory subscales of the AASP, SP, and SEQ. Pearson’s correlation coefficients and *p*-values are presented, along with Kendall’s ordinal equivalents; the latter are robust to outliers. *P*-values are given twice in each cell: above without correction and below with correction for ten comparisons per the Holm-Bonferroni procedure (separately for Pearson’s and Kendall’s correlations). | | | | |
| --- | --- | --- | --- | --- |
| Questionnaire Subscale | Pearson correlation | | Kendall’s correlation | |
|  | *r* | *p* | *τ* | *p* |
| AASP Auditory Low Registration | .19 | .35  >.99 | .21 | .15  >.99 |
| AASP Auditory Sensation Avoiding | .21 | .30  >.99 | .14 | .33  >.99 |
| AASP Auditory Sensation Seeking | .39 | .052  .52 | .35 | .02*  .20 |
| AASP Auditory Sensory Sensitivity | .08 | .68  >.99 | .09 | .52  >.99 |
| SP Auditory High Threshold | .33 | .08  .70 | .24 | .09  .78 |
| SP Auditory Low Threshold | .19 | .34  >.99 | .13 | .33  >.99 |
| SEQ Auditory HYPO | .04 | .83  .83 | .02 | .88  .88 |
| SEQ Auditory HYPER | −.30 | .10  .79 | −.21 | .10  .84 |
| SEQ Auditory SIRS | .18 | .32  >.99 | .18 | .18  >.99 |
| SEQ Auditory EP | −.16 | .38  >.99 | −.02 | .85  >.99 |

| Supplementary Table 4. Results of analyses conducted in the autistic group exploring correlations between somatosensory P60 ERP amplitudes and tactile subscales of the AASP, SP, and SEQ. Pearson’s correlation coefficients and *p*-values are presented, along with Kendall’s ordinal equivalents; the latter are robust to outliers. *P*-values are given twice in each cell: above without correction and below with correction for nine comparisons per the Holm-Bonferroni procedure (separately for Pearson’s and Kendall’s correlations). | | | | |
| --- | --- | --- | --- | --- |
| Questionnaire Subscale | Pearson correlation | | Kendall’s correlation | |
|  | *r* | *p* | *τ* | *p* |
| AASP Tactile Low Registration | .47 | .02*  .14 | .29 | .048*  .43 |
| AASP Tactile Sensation Avoiding | .34 | .09  .70 | .23 | .11  .88 |
| AASP Tactile Sensation Seeking | .07 | .73  >.99 | .04 | .81  >.99 |
| AASP Tactile Sensory Sensitivity | .04 | .85  >.99 | .09 | .52  >.99 |
| SP Tactile High Threshold | .00 | >.99  >.99 | .02 | .87  >.99 |
| SP Tactile Low Threshold | −.07 | .73  >.99 | −.04 | .76  >.99 |
| SEQ Tactile HYPO | −.02 | .92  >.99 | .04 | .78  >.99 |
| SEQ Tactile HYPER | .03 | .89  >.99 | .00 | .97  >.99 |
| SEQ Tactile SIRS | .01 | .96  >.99 | .00 | >.99  >.99 |
